# When inflammatory stressors dramatically change, disease phenotypes may transform between autoimmune hematopoietic failure and myeloid neoplasms

**DOI:** 10.3389/fimmu.2024.1339971

**Published:** 2024-02-15

**Authors:** Xi-Chen Zhao, Bo Ju, Nuan-Nuan Xiu, Xiao-Yun Sun, Fan-Jun Meng

**Affiliations:** ^1^ Department of Hematology, The Central Hospital of Qingdao West Coast New Area, Qingdao, Shandong, China; ^2^ Department of Hematology, The Affiliated Hospital of Qingdao University, Qingdao, Shandong, China

**Keywords:** aplastic anemia, myelodysplastic syndrome, acute myeloid leukemia, inflammatory stressors, leukemic transformation, antileukemic immunity

## Abstract

Aplastic anemia (AA) and hypoplastic myelodysplastic syndrome are paradigms of autoimmune hematopoietic failure (AHF). Myelodysplastic syndrome and acute myeloid leukemia are unequivocal myeloid neoplasms (MNs). Currently, AA is also known to be a clonal hematological disease. Genetic aberrations typically observed in MNs are detected in approximately one-third of AA patients. In AA patients harboring MN-related genetic aberrations, a poor response to immunosuppressive therapy (IST) and an increased risk of transformation to MNs occurring either naturally or after IST are predicted. Approximately 10%–15% of patients with severe AA transform the disease phenotype to MNs following IST, and in some patients, leukemic transformation emerges during or shortly after IST. Phenotypic transformations between AHF and MNs can occur reciprocally. A fraction of advanced MN patients experience an aplastic crisis during which leukemic blasts are repressed. The switch that shapes the disease phenotype is a change in the strength of extramedullary inflammation. Both AHF and MNs have an immune-active bone marrow (BM) environment (BME). In AHF patients, an inflamed BME can be evoked by infiltrated immune cells targeting neoplastic molecules, which contributes to the BM-specific autoimmune impairment. Autoimmune responses in AHF may represent an antileukemic mechanism, and inflammatory stressors strengthen antileukemic immunity, at least in a significant proportion of patients who have MN-related genetic aberrations. During active inflammatory episodes, normal and leukemic hematopoieses are suppressed, which leads to the occurrence of aplastic cytopenia and leukemic cell regression. The successful treatment of underlying infections mitigates inflammatory stress-related antileukemic activities and promotes the penetration of leukemic hematopoiesis. The effect of IST is similar to that of treating underlying infections. Investigating inflammatory stress-powered antileukemic immunity is highly important in theoretical studies and clinical practice, especially given the wide application of immune-activating agents and immune checkpoint inhibitors in the treatment of hematological neoplasms.

## General overview of aplastic anemia and myeloid neoplasms

Aplastic anemia (AA) is an acquired form of autoimmune hematopoietic failure (AHF). Traditionally, AA has been considered a benign hematological disease. AA pathogenesis results from the destruction of hematopoietic progenitor cells (HPCs) by deranged autoimmune responses that occur mainly in the bone marrow (BM) environment (BME). After an initial insult by pathogenic environmental challenges, such as genotoxic exposure or microbial infection, the activated immune responses perpetuate and result in immune-mediated impairment of blood cell production and subsequent clinical presentations of BM hypoplasia/aplasia and peripheral cytopenia in one or more cell lineages (1, 2). Currently, the diagnosis of AA is largely based on markedly decreased BM cellularity and hematopoietic volume with fatty replacement and the absence of overt dysplastic hematopoiesis in HPCs (3, 4). AA is a highly heterogeneous disease entity that converges on a pathophysiological process of persistently deranged autoimmune responses and heavily exhausted HPCs. Patients with AA exhibit varying clinical presentations regarding disease phenotype, disease severity, disease course, treatment response to immune suppressants, and the likelihood of transformation to myeloid neoplasms (MNs). This high heterogeneity is driven by the diversity of the constitutional genetic background, the competence of innate immune cells, the appropriateness of adaptive immune responses to environmental challenges, basic functions of other organ systems, events that guide immune responses, and perhaps most importantly, conditions that sustain deranged autoimmune reactions (1–4).

Myelodysplastic syndrome (MDS), myeloproliferative neoplasm (MPN), and acute myeloid leukemia (AML) are unequivocal MNs. MNs result from the acquisition and accumulation of somatic mutations in HPCs. Genetic damage is generated from the exposure of HPCs to various oncogenic agents, and the emergence of leukemic clones arises from the selective advantage of transformed HPCs in the context of chronic inflammatory BME. Oncogenic mutations in transformed HPCs lead to altered proliferation, self-renewal, and antiapoptotic capacity. The dysregulated growth of leukemic cells results in the overgrowth of their normal counterparts and the suppression of normal blood cell production, clonal hematopoiesis, and corresponding hematological presentations (5–8). The presence of dysplastic features indicates a diagnosis of MNs, and an increased percentage of myeloblasts is the most significant biomarker representing leukemic hematopoiesis and entry into advanced stages (9, 10). Immunological, cytogenetic, and biological molecular analyses can provide valuable information for diagnosis, classification, and risk stratification. MDS, MPN, and AML are also highly heterogeneous with somatic mutations involving hundreds of genes in association with their pathogenesis. Each somatic mutation plays a distinctive role in malignant transformation or affects clinical complications, hematological presentations, cellular components, immunological signatures, disease progression, and treatment response (5–8).

Accumulating evidence supports that there is an intrinsic relationship between AHF and MNs regarding their clonal hematopoiesis, immunological profile, and disease phenotypic transformations.

## Reciprocal transformations between AA and MNs

By definition, AA is distinct from MDS, MPN, and AML with differences in fundamental pathogenesis, biological behaviors, laboratory features, treatment strategies, and prognostic significance. However, disease phenotypes can transform between AHF and MNs. The natural transformation of AA to MNs has been widely investigated (11–14) and is generally ascribed to the acquisition and accumulation of oncogenic mutations due to selective pressure and immune exhaustion in the setting of a chronic inflammatory milieu (15–18). It is well known that antithymocyte globulin (ATG) plus cyclosporine-based immunosuppressive therapy (IST) promotes leukemic transformation. Approximately 10%–15% of patients with severe aplastic anemia (SAA) experience leukemic transformation following IST, and in some patients, this transformation emerges during or shortly after IST (19–23). Such a short interval that spans the transformation process indicates that leukemic clones preexist in the SAA stage (24–27) and rapidly expand due to depletion of cytotoxic T lymphocytes (CTLs) or the induction of immune tolerance (28–32) rather than due to the acquisition of novel molecular events. In patients with genetic aberrations typically observed in MNs, there is a greater frequency of transformation to MNs. Moreover, 20%–30% of SAA patients fail to respond to IST, and these patients have a significantly greater frequency of MN-related genetic aberrations compared to patients who achieve a good treatment response. Even in patients who achieve a hematological response, the presence of MN-related genetic aberrations is associated with a reduced sensitivity to IST, a low rate of deep remission, a high frequency of disease relapse, and a significantly increased risk of leukemic transformation (19–23).

**Figure 1:**
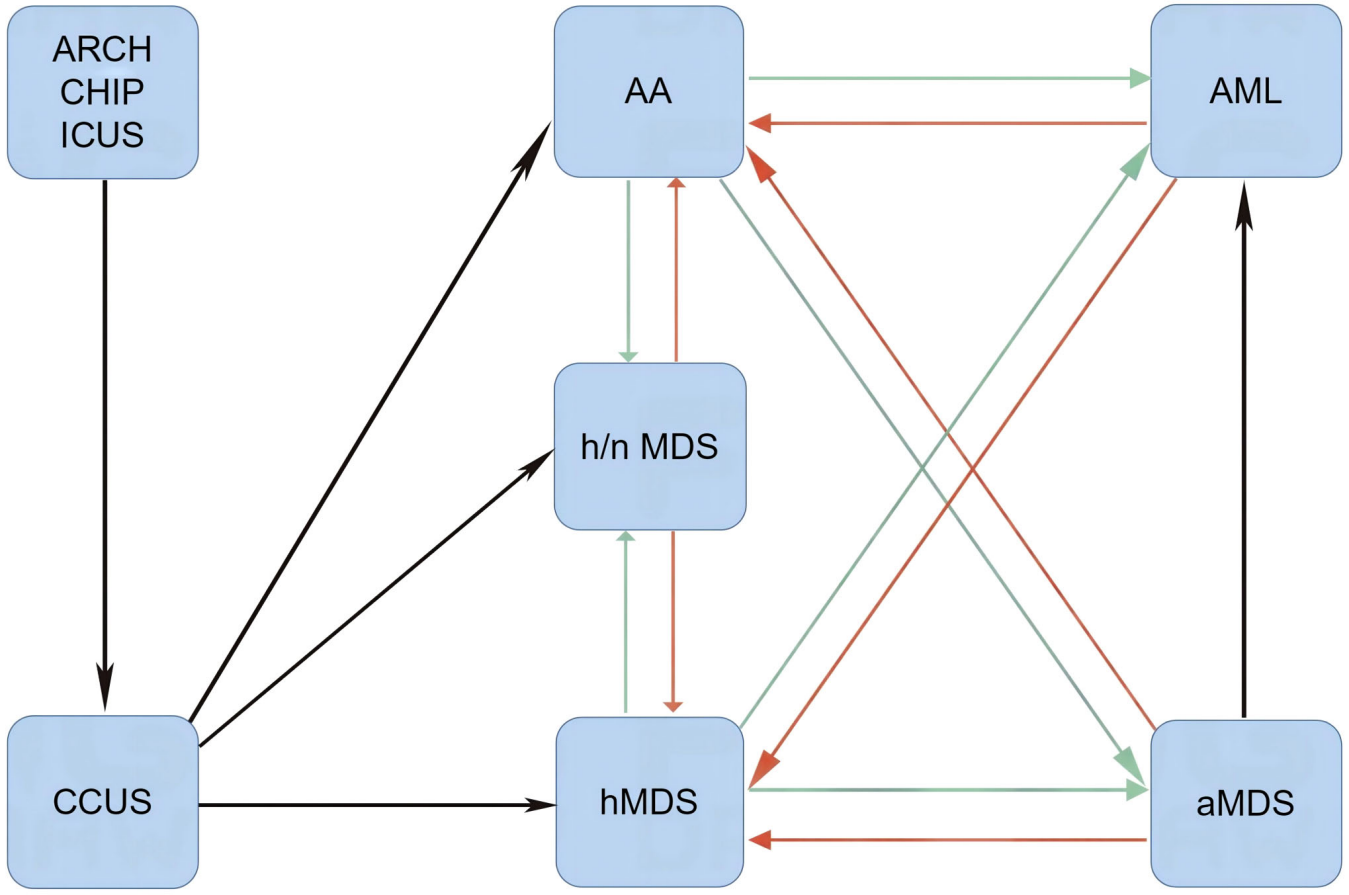
Disease phenotypic transformations between AHF and MNs. When Th1 responses are dramatically activated (red arrows) by immune-activating agents and/or immune checkpoint inhibitors, normal and leukemic hematopoieses are suppressed. In this setting, patients present with AA-like syndrome or hMDS. After removal of immune-activating agents or depletion of effector memory cytotoxic lymphocytes, mitigated Th1 responses lead to the reduction of inflammatory stress-powered antileukemic activities (green arrows), and symptomatic myeloid neoplasms may emerge. AA, aplastic anemia-like syndrome; aMDS, advanced myelodysplastic syndrome; AML, acute myeloid leukemia; ARCH, age-related clonal hematopoiesis; CCUS, clonal cytopenia of undetermined significance; CHIP, clonal hematopoiesis of indeterminate potential; hMDS, hypoplastic myelodysplastic syndrome; h/nMDS, hyper/normoplastic myelodysplastic syndrome; ICUS, idiopathic cytopenia of undetermined significance; AHF, autoimmune hematopoietic failure; MNs, myeloid neoplasms.

Leukemic transformation in SAA patients can also occur after successful treatment of underlying infections and is frequently separated by a short duration of hematological remission. A rapid increase in leukemic blasts is the representative presentation of leukemic hematopoiesis (33–35). Moreover, patients with definitively diagnosed MNs can develop an aplastic crisis during infectious episodes. During aplastic crisis, the cytological and immunological features resemble those of SAA with a regression of leukemic blasts (35, 36). It remains unclear why patients with a similar genetic landscape or even an identical genetic background exhibit variable disease phenotypes. Furthermore, the significance of disease phenotypic transformations has not been fully elucidated through basic research and clinical practice. It is possible that environmental factors, most likely changes in inflammation strength or depletion of effector immune cells, affect the disease phenotype and contribute to phenotypic transformations (as schematized in **Figure 1**).

## Clonal hematopoiesis in AA

With the wide application of next-generation sequencing (NGS) in the diagnosis, mutational analysis, and risk stratification of hematological diseases (37, 38), it has been shown that more than one-third of patients definitively diagnosed with AA carry genetic aberrations that are well-known driver mutations in MNs (19–27). Although the number and clone size of mutant genes in AA are much smaller than those in MDS and AML, the number and clone size of these mutant genes can increase in patients who respond to IST (20–22). Currently, AA is also known to be a clonal hematological disease. Clonal HPCs gain a selective proliferative and survival advantage over their normal counterparts in a chronic inflammatory setting and become the dominant hematopoietic components (24–27). Moreover, some patients with MDS or AML can present heavily reduced BM cellularity at initial diagnosis or during disease progression, and patients with low-risk MDS have an immunological landscape similar to that of SAA (39–43).

## Immune dysregulation and an inflamed BME in AHF

AA and hypoplastic MDS (hMDS) are typical forms of immune-mediated AHF, and they share similar cytological and immunological landscapes (39, 40). In AHF, autoimmune CTLs (43–45) and natural killer (NK)/NKT cells (46–48) become activated and expanded. Trained CTLs [effector memory CTLs (emCTLs)] and NK/NKT cells (effector memory NK/NKT cells) exhibit elevated killing activities (43–48), increased secretory capacities (49–51), and enhanced sensitivities to antigen stimulation (52, 53). The oligoclonal feature of expanded emCTL infiltrating the BM suggests the presence of chronic stimulation that is driven by an antigen from the BME (43, 50). The excessive production of hematopoietic inhibitory mediators (54–57) due to T helper type 1 (Th1) immune responses (58–60) plays a central role in AHF pathogenesis. Additionally, upregulated expression of proapoptotic molecules (61–63) and decreased telomerase activity (64, 65) accelerate the death of HPCs. Upregulated expression of Toll-like receptors (TLRs) and costimulatory molecules in response to chronic inflammation enhances sensitivity to antigen stimulation, propagates inflammatory responses, induces the production of reactive oxygen species, augments apoptotic signals, amplifies pyroptoptic cell death, and favors a selective survival advantage, which forms a positive feedback loop to cause ineffective hematopoiesis and sustain clonal proliferation (66–69). Moreover, the number and function of regulatory T cells (Tregs) and regulatory B cells (Bregs) are dramatically decreased (70–73), as is the downregulation of anti-inflammatory mediators (74–76). Defects in HPCs and dysregulation of immune responses are also present in complete remission following IST (77, 78). Autoimmunity targeting HPCs by autoantigen-specific lymphocytes (50, 59) attracts a variety of immune cells (79–81). Immune cell infiltration and activation create an immune-active BME (79–83), which forms an inflammatory background and may determine a BM-predominant autoimmune impairment (39–42). The BM-predominant autoimmunity is amplified by an additional antigenic stimulation (84–87). Th1 immune responses suppress the production of erythrocytes, granulocytes, platelets, and B lymphocytes (49, 55–57, 87), which results in clinical and laboratory features of AA and hMDS (3, 4, 9, 10).

The development of an immune-active BME can be achieved in several ways. 1) Pattern recognition receptors (PRRs) sense pathogen-associated molecular patterns (PAMPs), or antigen-specific CTLs target pathogenic antigens of microbes that can survive and smolder in BM cells (84–87). 2) Cellular components that cross-react with microbial epitopes are targeted by autoimmune lymphocytes trained in active infectious diseases (88), particularly inflammatory conditions involving the gastrointestinal tract (89, 90), which can continuously supply intestine-derived antigens from pathogenic or commensal microbes in the context of increased epithelial permeability (90–92). 3) Somatic mutations in epigenetic modulators or transcription factors lead to the excessive production of inflammatory cytokines (93, 94). 4) PRRs sense damage-associated molecular patterns (DAMPs) (95–98), or autoimmune CTLs target neoplastic antigens of genetic or epigenetic products (40, 59, 99–101) in transformed HPCs. If autoimmune responses target DAMPs or neoplastic antigens on leukemic HPCs, the role of autoimmune responses in AHF may be antileukemic (95, 97, 102).

## Contribution of inflammatory stressors to autoimmune diseases

### Chronic intracellular infections in AID pathogenesis

Chronic infectious diseases, especially intracellular infections, are closely associated with the development, persistence, and acceleration of autoimmune-related impairments in various organ systems (103–110). Autoimmune diseases (AIDs), which usually manifest as organ-specific impairments, are frequently localized at sites away from infectious niduses. The severity of AIDs commonly parallels the severity of chronic infectious conditions (111, 112), which provides persuasive evidence that chronic infections contribute to the perpetuation and acceleration of autoimmune responses. Low-affinity autoimmune lymphocytes can escape the process of clonal selection and clonal deletion (113–116), and the development of AIDs critically requires the engagement of environmental factors to disturb immune homeostasis and generate high-affinity autoimmune lymphocytes by somatic mutations in the antigen-binding region (affinity mutation) (117–119).

### Mechanisms by which infectious diseases induce AID pathogenesis

Several hypotheses have been proposed to explain the relationship between the occurrence of infectious diseases and the generation of autoimmune responses. 1) When infected by microbes or inoculated with vaccines, hosts generate high-affinity antibodies or emCTLs through class switching and affinity maturation in the fight against invading pathogens. High-affinity antibodies or emCTLs cross-react with epitopes of autoantigens in certain tissues (molecular mimicry) (120–123). After being trained in response to microbial infections, these autoreactive lymphocytes migrate to remote organs, exert an immunological attack, and induce tissue damage when recognizing cognate autoantigens (117–119). 2) When tissue is infected by pathogenic microbes, autoreactive lymphocytes in the inflamed niche can be activated and trained by antigen-presenting cells and autoantigens, which form high-affinity autoimmune B and T cells and exert an immunological attack on corresponding tissues (bystander activation) (124–126). 3) In the inflamed niche, cryptic epitopes can be exposed, recognized, and presented to immune cells as exotic antigens to elicit immunological reactions. This immunological attack on cryptic epitopes sustains inflammatory impairment in affected tissues (epitope spreading) (127, 128). 4) During chronic infections, antigens or antibodies can be modified, frequently by glycosylation, which results in altered antigenicity of tissue components or an altered affinity of autoantibodies (posttranslational modifications) (129–132). 5) Certain components of infecting microbes can activate polyclonal lymphocytes and result in the generation of self-reactive antibodies and autoimmune CTLs that can bind to tissue components. These microbial components are called superantigens. Superantigen-induced self-reactive antibodies or autoimmune CTLs initiate an immunological attack (superantigen stimulation) (133–135). The same microbes can trigger AIDs in different organ systems through different mechanisms, and AIDs in certain organs can be induced by different microbial infections through different mechanisms.

After pathogenic antigens have been cleared, the host immune system rapidly returns to a homeostatic state by complex immune regulatory mechanisms to limit inflammatory damage (136–138). However, effector memory lymphocytes have the ability to respond promptly and play a major role when the host encounters the same or cognate epitopes (139–141). Chronic infectious diseases can continuously supply pathogenic antigens or create an inflammatory milieu in which host immune cells persistently produce inflammatory cytokines (84–92) and thereby synergistically sustain the activated form of host autoimmune cells in cognate antigen-dependent or antigen-independent pathways (139–141) to attack antigen-specific cells and innocent bystander tissues (142, 143).

### Gut inflammatory conditions in AID pathogenesis

In addition to chronic intracellular infections, the gastrointestinal tract is another common chronic inflammatory nidus. As an important organoid in the human body (144, 145), the gut microbiota plays an essential role in the stimulation, education, and maturation of the host immune system and is the most important element that dynamically affects host immune homeostasis throughout life (146–148). Indeed, host peripheral lymphatic tissues develop poorly in the absence of the gut microbiome (149–151). The associations of AIDs with gut dysbiosis and gut inflammatory conditions have been investigated extensively (90, 92, 147, 152, 153). Patients with inflammatory bowel disease (IBD) have a high frequency of extraintestinal autoimmune manifestations including systemic connective tissue diseases, seronegative spondyloarthropathy, systemic vasculitis, and organ-specific autoimmunity in different organ systems (154, 155).

The critical requirement of the gut microbiome for inducing and sustaining autoimmunity in genetically susceptible animal models has been verified in multiple AIDs and provides affirmative evidence for the contribution of gut dysbiosis and gut inflammatory disorders to the development of autoimmune pathogenesis. These animal models spontaneously develop AIDs under conventionally reared conditions, but autoimmune impairment does not occur in germ-free or specific pathogen-free conditions (155–161). In animal models of AIDs, the clearance of the gut microbiome can effectively alleviate disease severity (157). Even in conventionally reared animals, administration of probiotic antibiotic agents can significantly delay disease penetration (162). This is not surprising because the gastrointestinal tract harbors the most abundant lymphoid tissues and microbial community (163, 164). The microbial community and gut homeostasis are constantly subject to various environmental challenges (165, 166), including pathogen intruders (167–170), chemical damage (171, 172), drug exposure (173–175), and nutrient delivery (176–178). Harmful challenges can compromise the integrity of the intestinal epithelium, induce dysfunction, cause dysbiosis, and trigger gut inflammatory disorders; these can occur not only by injuring diseased segments but also by affecting other segments through disruption of microbial ecology (179–181). Gut dysbiosis can initiate immune responses and cause inflammatory lesions in the gastrointestinal tract (181–184). In the setting of a compromised intestinal structure, impaired barrier functions allow host immune cells to come into intimate contact with intestine-derived antigens (89–92). This occurs not only from pathogenic agents but also from commensal microbes and undigested food (185–187) and thereby activates host immune cells, disturbs immune homeostasis, overrides immune tolerance, and triggers deranged immune responses (188–192).

The gastrointestinal tract can continuously supply a large amount of intestine-derived antigens and persistently activate a substantial number of immune cells. The structural and functional features of the gastrointestinal tract render the organoid vulnerable to be an active inflammatory nidus in which host immune homeostasis is disturbed and inflammatory stresses are sustained with sufficient inflammation strength to perpetuate autoimmune responses in remote organ systems (163, 164). In this process, probiotic commensal microbe-derived metabolites play important roles in maintaining epithelial integrity, regulating host immune responses, and modulating autoimmunity (177, 178, 193–196). An inadequate dietary supply can induce gut dysbiosis and immune dysregulation and affect epithelial repair function (197–200). Chronic gut inflammatory disorders likely work as amplifiers linking systemic immune dysregulation with environmental factors to augment the intensity of extraintestinal autoimmune responses and thereby power autoimmune impairment (201–203). Gut microbiota also plays an indispensable role in combating intracellular infections (204–206) and in eliciting anti-infection responses through vaccine inoculation (207, 208), which reinforces the role of gut microbiota in amplifying systemic immune activities.

### Non-specific antigen stimulation in AID pathogenesis

Autoimmune impairments in genetically predisposed animals can also be initiated by adjuvant agents, PAMPs, recombinant inflammatory cytokines, and immune checkpoint inhibitors (ICIs). The Bacillus Calmette–Guerin (BCG) vaccine has long been recognized in the induction of AIDs (209, 210), and Freund’s adjuvant has been widely used in experimental models to trigger autoimmunity (211, 212). Autoimmune responses are common complications that occur following not only BCG but also other inoculations. This leads to the creation of the specific term “autoimmune syndrome induced by adjuvant (ASIA)” (213–216). Polyriboinosinic acid:polyribocytidylic acid (polyI:C) (217, 218), endotoxins (219–221), and fungal peptidoglycans (222–224) activate different PRRs. These PAMPs can trigger and sustain immune derangement by disrupting immune homeostasis (225, 226). ICIs (227, 228) and even hematopoietic growth factors (229, 230) can promote the penetration of AIDs. The initiation of AIDs by non-specific antigen stimulation suggests that systemic inflammatory stressors, rather than specific antigen stimulation, can synergize with a primary immune-active background to trigger deranged organ-specific autoimmune processes (125, 126, 139).

## Potential antileukemic mechanism in AHF

In 2009, Nissen (231) proposed that autoimmune responses occurring in acquired AA may function as an antileukemic mechanism against malignant proliferation. In the SAA stage, the decreased number and function of Tregs and Bregs, the decreased production of inhibitory cytokines, the reduced expression of coinhibitory molecules, the increased Th1 responses, the increased number and function of CTLs and NK/NKT cells, the increased antigen-presenting activities, and the excessive expression of proapoptotic and costimulatory molecules on HPCs may represent an adaptive process in response to ongoing antigen stimulation. Along with disease progression, the immunological signature in the BME is transformed from a Th1-predominant response to an immune exhaustion state in which the function of infiltrated autoimmune lymphocytes is inhibited and immune surveillance is gradually dampened in adaptation to the chronic inflammatory milieu. The immune exhaustion state favors leukemic cell survival and facilitates the penetration of symptomatic MNs (15–18). The extrapolation was mainly drawn from tumor regression and concomitant autoimmune phenomena in diseased organs during the treatment of non-hematological malignancies with immunological therapy. The author also described examples of hematological neoplasms in which leukemic cell regression was observed along with the appearance of prolonged aplastic cytopenia and autoimmune reactions (AA-like syndrome) after intensive chemotherapy or other treatment modalities. The author coined the phrase “pancytopenia of unknown significance” to describe this clinical phenomenon. Severe BM hypoplasia, systemic inflammatory symptoms, increased autoreactive CTLs, and decreased Tregs were observed in this AA-like syndrome. These cytological and immunological features indicate that antileukemic immunity has been reinvigorated.

**Figure 2:**
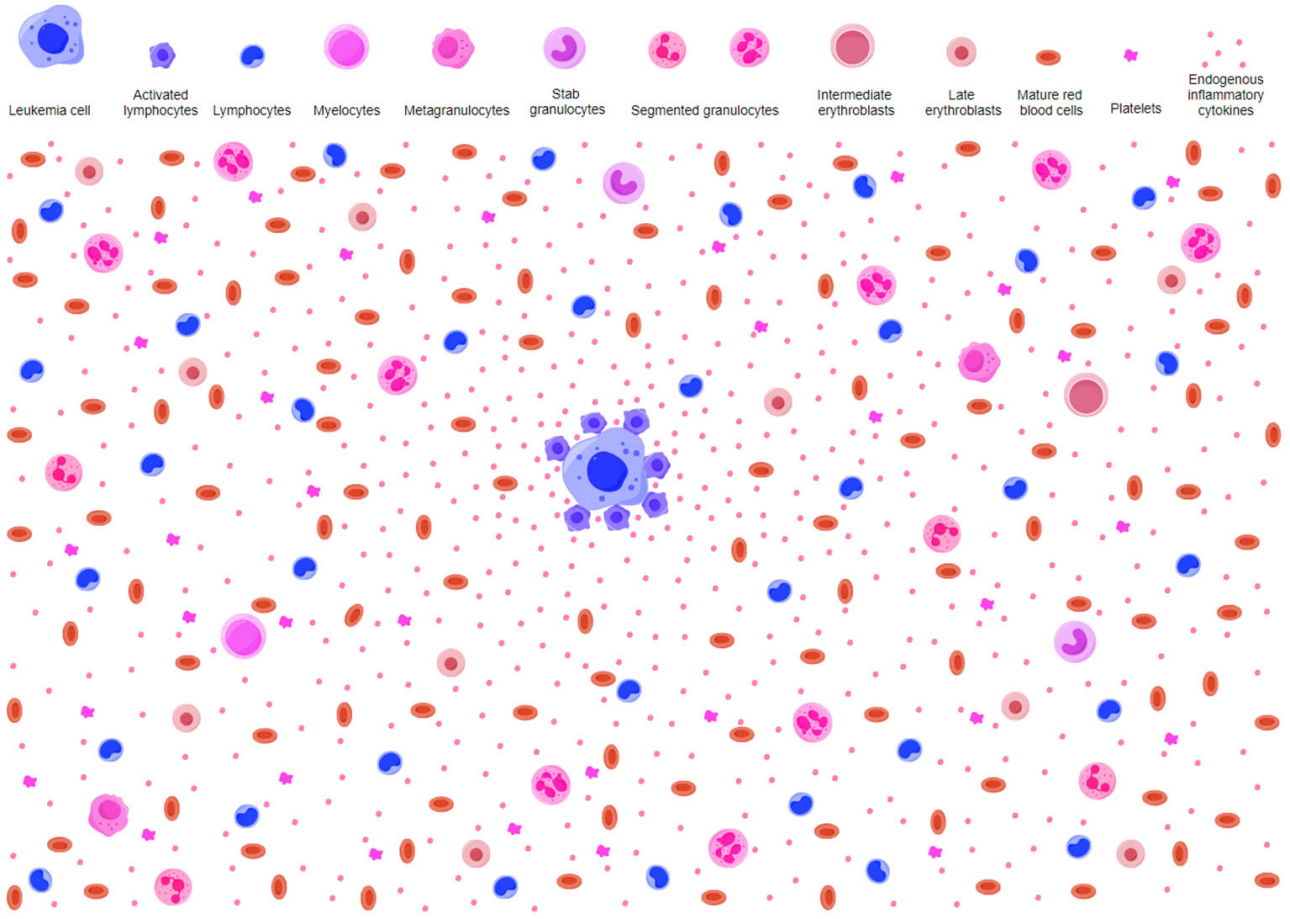
Potential antileukemic mechanism in AA-like syndrome. AA-like syndrome is characteristic of reduced HPCs and relatively increased lymphocytes without overt dysplasia. Early hematopoietic progenitors are rare. AA-like syndrome is caused by host immune surveillance against transformed HPCs. Immune responses to neoplastic molecules on leukemic HPCs activate innate and adaptive immune cells. Activated lymphocytes secrete proinflammatory cytokines and create an inflammatory background in the BME. Normal HPCs are suppressed by hematopoietic inhibitory mediators as innocent bystander victims in the process of host immune cells attacking leukemic HPCs. Because of the preferential suppressive effects on transformed HPCs, myeloblasts are hardly observed. The small number of nucleated hematopoietic cells and more intensive responses to transformed HPCs make it difficult to identify overt aplasia, which results in a diagnosis of AA in most circumstances. AA, aplastic anemia; HPCs, hematopoietic progenitor cells; BME, bone marrow environment.

Indeed, AHF can occur in response to neoplastic antigens or DAMPs (95, 96, 102, 232). The acquisition of autoantibodies against the neoplastic antigen carbonic anhydrase I induces AA-like syndrome and leukemic cell regression (102). Constitutive activation of the RAS-MAPK pathway can induce HPC senescence (96), and the absence of PRRs that sense neoplastic molecules results in accelerated leukemia cell proliferation (97). Similar to solid tumors (233–235), immune responses to neoplastic antigens (99–102) and stress in response to HPC damage (95–97) establish a chronic inflamed BME (93, 94, 236, 237). The inflamed BME enhances sensitivity to exogenous antigen stimulation (52, 66, 67) and accelerates apoptotic death in leukemic (95, 96, 238, 239) and normal (95, 96, 142, 143) HPCs, which are the common clinical and laboratory features of AA and low-risk MDS (39, 40, 49, 240) (as schematized in **Figure 2**).

Antileukemic immunity-induced Th1 responses preferentially repress leukemic cells and their normal counterparts, which can lead to pronounced neutropenia (42, 102, 232). Autoimmune neutropenia can persist for many years prior to the emergence of symptomatic MNs. In this stage, the BM is hypocellular, and cytological and immunological features exhibit immune-mediated hematopoietic impairments in the absence of morphologically identifiable leukemic blasts (241, 242). A significant proportion of MDS patients with overt dysplasia present with hypocellular BM and peripheral pancytopenia at initial diagnosis or during disease progression and these patients frequently have longer survival (21, 23, 42, 240). Prolonged neutropenia after intensive chemotherapy has been associated with deep remission, longer remission duration, reduced frequency of relapse, and restoration of Th1 immune responses (243, 244). Substantial evidence supports an antileukemic role of autoimmune responses in AHF (42, 95, 102, 232), at least in a large number of AHF patients who have MN-related genetic aberrations (19–26). However, factors underlying the exaggerated autoimmune responses that influence antileukemic activities and disease phenotypes were not discussed in Nissen’s manuscript (231).

## Inflammatory stressors power antileukemic immunity

**Figure 3:**
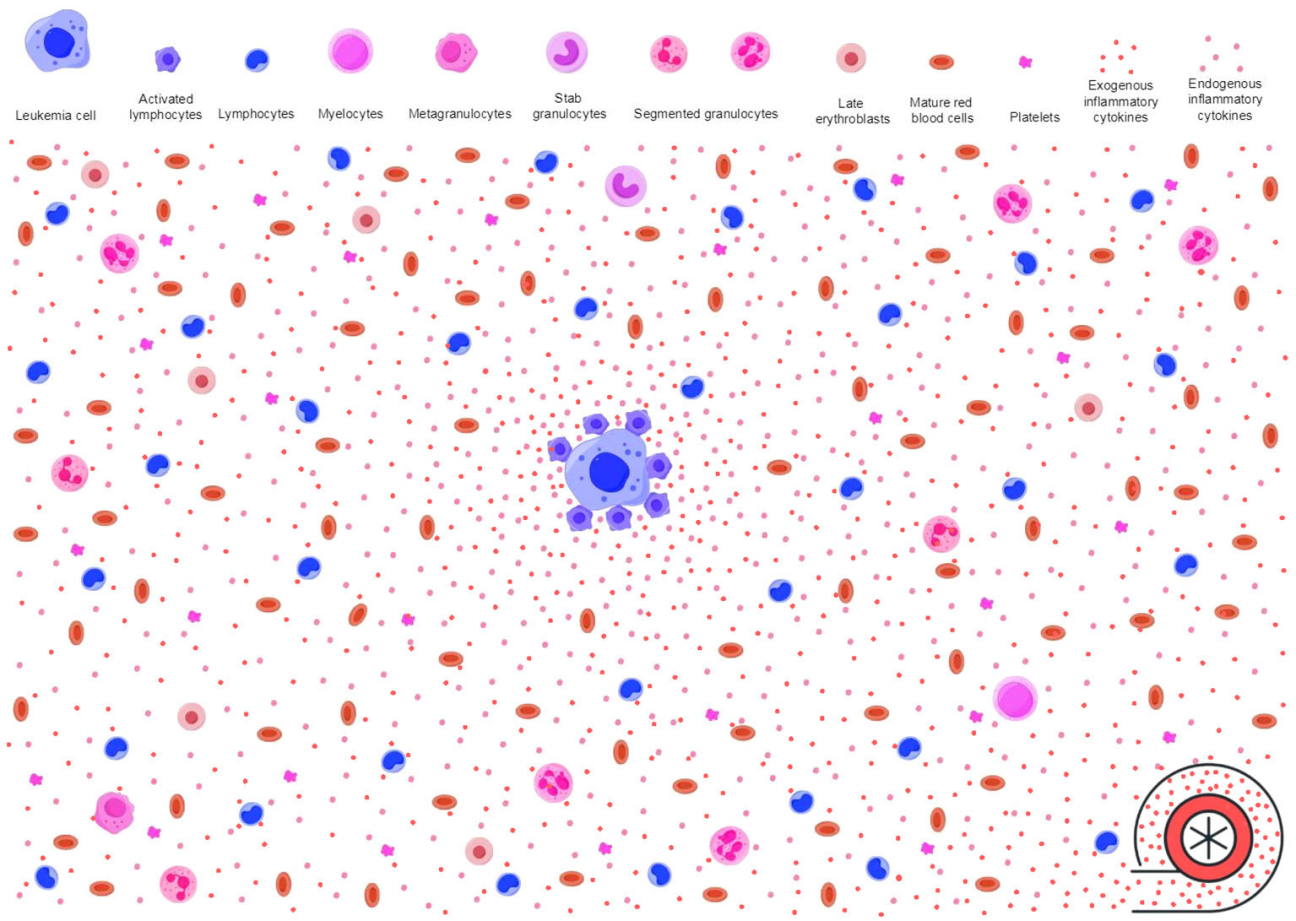
Inflammatory stressors power antileukemic activities. As described in **Figure 2**, immune surveillance in response to neoplastic molecules releases proinflammatory cytokines (endogenous inflammatory cytokines as shown in pink dots) and creates an inflammatory background. However, the baseline antileukemic immunity may not be sufficient to effectively suppress leukemic HPCs. Immune responses to invading microbes produce additional activated immune cells and proinflammatory cytokines (exogenous inflammatory cytokines as shown in red dots). These pathogen-activated immune cells and the proinflammatory cytokines migrate to the inflamed BME through blood circulation, power the primary inflammatory milieu, and enable the antileukemic immunity to achieve an intensity that is sufficient to effectively suppress leukemic HPCs. The primary inflamed BME and extramedullary inflammatory stressors synergistically trigger and sustain AA-like syndrome. HPCs, hematopoietic progenitor cells; BME, bone marrow environment; AA, aplastic anemia.

The transformation between AHF and clinically overt MNs (MDS with excessive blasts or AML in which an increased percentage of myeloblasts is the most representative parameter in determining leukemic hematopoiesis) can reciprocally occur. The switch that shapes the disease phenotype is a change in the strength of extramedullary inflammatory conditions (33–36). Extramedullary infectious diseases have a conspicuous impact on phenotypic presentations. During active inflammatory episodes, normal and leukemic hematopoieses are simultaneously suppressed with regression of leukemic blasts on cytological examination and Th1-predominant responses on immunological analysis. Aplastic cytopenia, the disappearance of leukemic blasts, Th1-predominant responses, and a small number of nucleated blood cells for identification of dysplasia result in cytological and immunological features that meet the criteria for the diagnosis of AA (33–36). Following successful control of the inflammatory episodes and a short duration of hematological remission, quickly expanded leukemic clones lead to the appearance of substantial leukemic blasts and symptomatic MNs (33–35). Patients with clinically overt MNs can develop an aplastic crisis during active inflammatory episodes in which systemic inflammatory stress is dramatically aggravated (35, 36). Within this period, the BM becomes aplastic/hypoplastic, and leukemic blasts disappear with the infiltration of autoimmune lymphocytes. These phenomena indicate that autoimmune responses in AHF are an inflammatory stress-powered antileukemic mechanism. Immune surveillance against leukemic blasts contributes to BM-specific autoimmune impairments, while inflammatory stressors strengthen autoimmune responses (as schematized in **Figure 3**).

This process simulates spontaneous remission in AML patients after an infectious episode and aplastic crisis (245–252). The mechanism is presumed to be due to reversion of the immune exhaustion state and restoration of antileukemic immunity through activation of immune cells and their secretion of proinflammatory cytokines in response to invading pathogens (250–253). Another frequent situation that induces spontaneous remission is that patients with hypoplastic AML (hAML) are treated with hematopoietic growth factors. To date, spontaneous remission has been reported in more than 200 AML patients. The remission duration usually lasts for 2–6 months, which indicates that leukemic clones cannot be eradicated even under inflammatory stress-powered antileukemic immunity (245, 254). This may be due to the low expression of neoplastic antigens on dormant leukemic cells (255–258). However, repeated spontaneous remissions occur in a paucity of patients (245, 247), which suggests that patients do not completely lose the hematopoietic regulatory mechanisms and the inflammatory stress-powered antileukemic immunity. Another interesting phenomenon also suggests the existence of inflammatory stress-powered antileukemic activities (243, 244). Prolonged neutropenia due to hematopoietic suppression in AML patients after intensive chemotherapy is usually complicated by durable or repeated infectious episodes, which frequently result in death. If patients survive prolonged neutropenia, they may achieve deep remission, longer remission duration, and a reduced frequency of relapse. Recombinant type I inflammatory cytokines (259–262), interferon inducers (263–265), and BCG inoculation (266–268) have been used to treat hematological and non-hematological neoplasms and have shown efficacy in the control of tumor growth or even the clearance of malignant clones. Other vaccine inoculations that can stimulate Th1 responses have also been shown to have antitumor effects (269–271). These non-specific immunological therapies and their treatment effects indicate that inflammatory stressors, rather than antigen-specific responses to invading pathogens, augment antitumor activities (139).

## Reversion of the immune-exhausted state by Th1 responses and ICI treatment

Immune exhaustion in adaptation to persistent inflammatory stimulation is a common mechanism underlying the escape of leukemic clones from immune surveillance (17, 18). Hyporesponsiveness is characterized by a high infiltration of negative immune cells, excessive secretion of anti-inflammatory cytokines by immune regulatory cells, and high expression of immune checkpoint molecules on tumor cells in the tumor microenvironment, which work to limit the magnitude of inflammatory responses, avoid excessive tissue damage, and maintain a dynamic immune balance in the context of chronic inflammatory stressors (272, 273). In an immune exhaustion state, dampened antileukemic immunity favors leukemic cell escape from immunological attack and facilitates the penetration of symptomatic MNs. ICIs block coinhibitory molecules and reinvigorate antitumor immunity. ICIs have been successfully used to treat MNs (273–275), and a high-grade inflammatory profile in the BME is a predictor of a good therapeutic response to ICI treatment (276, 277) and suggests that antileukemic immunity is closely related to an inflamed BME. The relationship between an inflamed microenvironment and ICI treatment response has been more thoroughly studied in solid tumors (234, 278–280).

It is widely accepted that dysbiotic gut microbiota plays an important role in promoting tumor cell escape from immune attack in solid tumors (281–283), and the response to ICI treatment is strongly influenced by the abundance and diversity of the gut microbiome (284, 285). This also indicates that antitumor immunity necessitates the engagement of additional antigenic stimulation for immune surveillance against neoplastic antigens. A reduced gut microbial abundance and diversity due to antibiotic treatment can dramatically dampen ICI treatment responses (286). Modulation of the gut microbiota can reverse the dampened responses (287–290). Some pathogenic (291–295) and commensal (296, 297) microbes can stimulate Th1 immune responses and thereby power antitumor immunity (298–300) and enhance ICI treatment responses (301–303). One of the major adverse effects of ICI treatment is hematological toxicity (304–306), especially when treating hematological malignancies and when combined with other antileukemic agents (306). This reinforces the role of Th1 responses in AHF pathogenesis and inflammatory stress-related antileukemic immunity.

**Figure 4:**
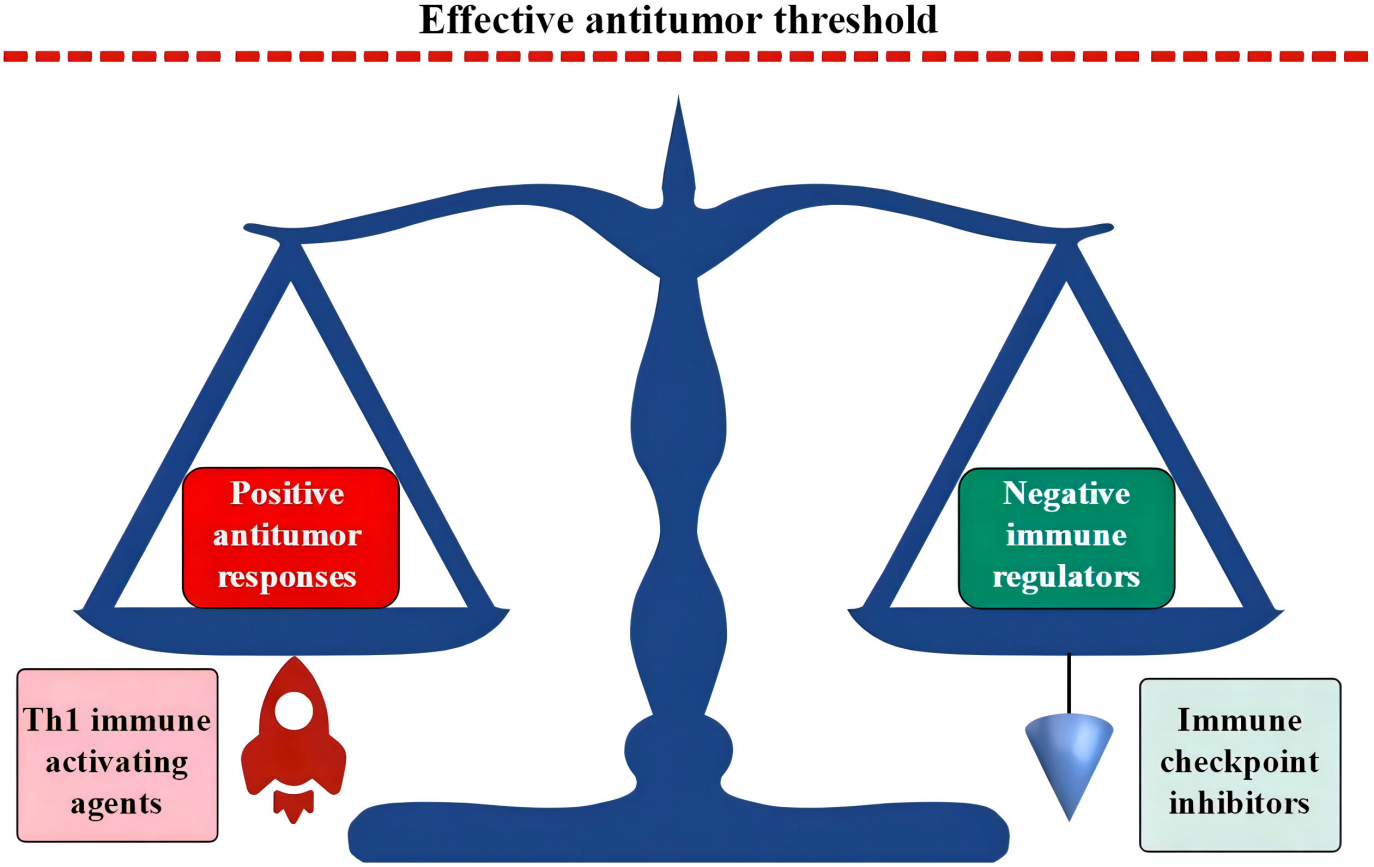
Th1 responses and ICI treatments break the dynamic balance in immune exhaustion state. In the immune exhaustion state, a novel dynamic balance has been established by the high expression of negative immune regulators in adaption to an inflamed BME. Activated Th1 responses propel positive antileukemic immunity, while ICI treatments block coinhibitory molecules. Both can break the vulnerable immune balance and induce positive antileukemic immunity. ICIs in combination with immune-activating agents may synergistically upregulate positive antileukemic immunity with an intensity that is able to override the threshold of effective antileukemic activities. ICI, immune checkpoint inhibitor; BME, bone marrow environment.

Blood cells are immune cells, and their production is regulated largely in response to microbial invasion. Therefore, hematopoietic cells are more sensitive to inflammatory mediators. When confronting an acute and limited infection, HPCs sense microbial antigens and inflammatory cytokines. With the pathogenic stimulation, host hematopoiesis proliferates and polarizes the differentiation toward the production of effector immune cells to fight invading microbes and replenish the consumed innate immune cells (307–310). However, chronic inflammatory stress in response to persistent intracellular infections can induce Th1-predominant immune responses and exhaust host hematopoietic functions. These responses predominantly exhaust granulopoiesis, erythropoiesis, megakaryocytopoiesis, and B lymphopoiesis (311–316) and result in the morphological and immunological signatures shown in AHF (3, 4, 10, 240). The upregulated expression of HLA-DR, TLRs, and the Nlrp3 inflammasome due to the primary immune-active BME increases sensitivity to antigen stimulation (50, 52, 66, 67) and apoptotic death of HPCs (50, 62, 63). In combination with extramedullary inflammatory stressors, the immune-active BME leads to BM-specific and inflammatory stress-powered autoimmune responses. In the absence of an inflamed BME, some infectious diseases, although able to induce Th1 immune responses, may not seriously injure hematopoietic function. Evidence for this is that patients with systemic rheumatic diseases are seldom complicated by AHF. Along with the persistence and exacerbation of inflammatory stressors, the host immune system can be impaired, which changes the immunological landscape from the proinflammatory phenotype of AHF to the immune exhaustion phenotype of advanced MN (317–320). Exhausted immune responses and DNA damage occurring under chronic inflammatory conditions are considered major contributors to the immune escape of leukemic cells (15, 16, 321, 322). The role of immune exhaustion in advanced MN may cause an increased threshold for effective antileukemic immunity (323). Enforcing an additional antigen stimulation or administrating ICIs can reverse the immune exhaustion state and thereby overcome the raised threshold and restore missed antileukemic immunity (35, 95, 250, 253, 323). Combination of immune-stimulating agents and ICI treatments may further potentiate antitumor activities (as schematized in **Figure 4**).

From this point of view, AHF and MNs have complex intrinsic relationships, and some patients with definitively diagnosed AA may be hMDS (39, 40, 324, 325) or hAML (102, 232). The absence of overt leukemic hematopoiesis in AHF likely results from the small number of nucleated cells available for identification of aplasia and leukemic blasts due to severe BM hypoplasia and a preferential attack on transformed HPCs by autoimmune CTLs and their secretion of hematopoietic inhibitory mediators (238, 239, 259–262). Indeed, neutropenia is a common preleukemic presentation in MNs, and in a significant proportion of MDS patients, neutropenia can be a solitary hematological abnormality that persists for many years (241, 242). Furthermore, a poor response to hematopoietic growth factors predicts a high frequency of disease progression (242). A decreased white blood cell count and concealed leukemic clones make it difficult to distinguish this AA-like syndrome from genuine AA (39, 40, 324, 325), which leads to a diagnosis of AA in most circumstances (3, 4), especially before the wide application of NGS in the diagnosis and stratification of hematological diseases.

## Decreased inflammatory strength facilitates leukemic transformation

AHF is an organ-specific AID. Autoimmune responses occur predominantly in the BM and result in the exhaustion of HPCs and impairment of mature blood cell production (1, 2, 39, 240). AA and hMDS are clonal hematological diseases (5, 25, 27, 240). Genetic aberrations in clonal hematopoiesis affect cell proliferation, self-renewal, and apoptosis (5–8). While hMDS is an unequivocal MN, AA has a high frequency of transformation to MNs. The natural evolution from AA to MNs is usually a long-term process (11–14) and is frequently attributed to genetic damage or immune exhaustion due to an inflamed BME (15–18). However, leukemic transformation can occur during or shortly after IST (19–23). Effective treatment of underlying infections can also induce leukemic transformation (33–35). Prior to the emergence of symptomatic MNs, patients usually experience a short duration of hematological remission. Leukemic transformation shortly after IST or treatment of underlying infections may better reflect the intrinsic relationship between AHF and MNs with regard to the influence of the host immunological state on the disease phenotype. This is because IST and treatment of underlying infections can rapidly change the host immune state without inducing novel genetic damage. An aplastic crisis with leukemic cell regression in definitively diagnosed MNs during inflammatory episodes provides a more persuasive argument for the influence of the host immune state on the disease phenotype (35, 36).

### How should we interpret BM-predominant autoimmune impairment in AHF when considering the very high prevalence of chronic inflammatory disorders in the gastrointestinal tract and other organ systems?

A reasonable explanation is that, similar to the microenvironment in solid tumors (233, 234, 272, 279), primary autoimmune pathogenesis targets specific antigens or DAMPs on HPCs or other blood cells in the BM, regardless of whether the targeted antigens are infectious (84–87) or neoplastic (95–102). Interactions between autoimmune CTLs and targeted antigens (50, 59, 100, 232) or between innate immune cells and DAMPs (95–98) evoke an inflammatory background in the BME (27, 93, 94, 236), which determines the BM-predominant autoimmunity. Defects in HPCs (77) and derangement of immune responses (78) are present not only in active SAA but also in hematological remission following IST, which suggests that intrinsic abnormalities in HPCs are responsible for the primary autoimmune response. Like patients with AA (326–331), patients with MDS and AML have also been shown to have a high frequency of autoantibodies that can bind erythrocytes, erythroblasts, and platelets (42, 99–102, 258), and autoantibodies against hematopoietic progenitors can directly induce Th1 immune responses (100, 102, 332, 333). Patients with these autoantibodies frequently present with hypoplasia and absence of dysplasia, which resembles a clinical phenotype observed in patients with AA (21, 42, 102).

### How should we interpret leukemic cell regression in the SAA stage and their dysregulated proliferation after the successful control of underlying inflammatory stressors?

A reasonable explanation is that targeted molecules are present in leukemic blasts (50, 59, 88, 100, 102). Neoplastic epitopes from genetic or epigenetic products of transformed HPCs (42, 93, 95, 97, 100, 232) are likely the targeting molecules responsible for the primary inflammatory BME. However, effective repression of leukemic blasts also requires the engagement of additional inflammatory stressors that are strong Th1 responses for the sustenance of antileukemic immunity (95, 161, 245, 246, 250). This is perhaps due to the low immunogenicity of neoplastic antigens on leukemic cells (255–258). Additional inflammatory stressors may play a role in sustaining a systemic inflammatory state (201–203), which together with the primary inflammatory BME creates an inflammatory niche with an intensity sufficient to repress normal and leukemic hematopoieses and synergistically forms the characteristic immunological signature in AHF. Th1 responses repress granulopoiesis, erythropoiesis, megakaryocytopoiesis, and B lymphopoiesis (311–317) in accordance with the cytological signature in AHF (3, 4, 10, 240). Th1 responses from extramedullary infectious diseases may preferentially strengthen antileukemic immunity in these lineages. ICIs for the treatment of hematological and non-hematological malignancies may also benefit from inflammatory stress-powered antitumor immunity. The administration of ICIs reverses the immune exhaustion state, promotes the infiltration of proinflammatory immune cells into the tumor microenvironment, and reduces the threshold for inducing effective antileukemic immunity (272–275). Inflammatory strength in the tumor microenvironment predicts the treatment response to ICIs (276–280). However, effective antitumor immunity requires additional antigen stimulation especially from the gastrointestinal tract (281–285), and the dynamic microbial ecosystem is the most important environmental factor for maintaining host immune homeostasis (146–148). Some pathogenic (291–295) and commensal (296, 297) microbes can stimulate Th1 responses, enhance ICI treatment-induced antitumor immunity (301–303), and even directly overcome the immune exhaustion state (298–300). This mechanism may play a more important role in MNs because blood cells are immune cells and their production is regulated largely in response to pathogen invasion (307–310). During active inflammatory episodes, TH1 immunity in response to invading pathogens powers antileukemic effects, which results in the regression of leukemic clones (33–36). After successful control of inflammatory diseases, suppressive effects on leukemic clones are mitigated and result in the penetration of morphologically identifiable leukemic hematopoiesis (35).

### How should we interpret the very short interval spanning the leukemic transformation process when considering that the development of symptomatic MNs through the acquisition and accumulation of novel oncogenic mutations is unlikely within such a short interval?

A reasonable explanation is that leukemic clones preexist but are repressed in AHF stages (19–27) and rapidly expand after the resolution of inflammatory stressors (95, 245, 250, 254). The persistence of autoantibodies (42, 99, 100, 258, 330) and autoimmune responses (59, 78, 93, 273, 321) in AA, MDS, and AML suggests that autoantigens are persistent and their immunogenicity is less intensive. Leukemic clones cannot be eradicated even in inflammatory stress-powered antileukemic immunity, which is perhaps due to the low expression of neoplastic antigens and immune molecules in dormant leukemic cells (35, 245, 250, 254). During an inflammatory episode against invading pathogens that can stimulate strong Th1 responses, a patient’s immune system is mobilized, and the vulnerable immune balance in adaptation to chronic inflammation is disrupted, which results in the recall of antileukemic immunity and concealment of leukemic blasts. This process is similar to that observed in spontaneous remission in AML patients after an infectious episode (35, 245, 250). During infectious episodes, patients demonstrate positive immune responses and frequently experience an aplastic crisis. After successful treatment of underlying infections, dampened inflammatory stress-powered antileukemic immunity leads to the expansion of previously suppressed leukemic clones and facilitates the appearance of symptomatic MNs, which is distinct from the naturally occurring clonal evolution in AA (11–14) due to the acquisition of novel somatic mutations and immune exhaustion in the chronic inflammatory milieu (16, 17, 321, 323).

### How should we interpret the short duration of hematological remission before the emergence of symptomatic MNs?

Prior to the emergence of symptomatic MNs, whether following IST (19–23) or effective treatment of underlying infectious diseases (33–35), patients experience a short duration of hematopoietic remission. A reasonable explanation for this short remission duration is that inflammatory stress-powered antileukemic activities can simultaneously repress normal (54–57) and malignant (245–254) hematopoiesis, which results in BM hypoplasia/aplasia with the infiltration of autoimmune lymphocytes and an increase in inflammatory intensity (34, 35). The absence of leukemic blasts may be due to the preferential repression of malignant proliferation (238, 239, 259, 260) and the small number of nucleated blood cells available for the identification of dysplastic features (232, 240, 324, 325). Following the effective treatment of underlying infections, inflammatory stress-mediated antileukemic activities are mitigated, which leads to the simultaneous proliferation of normal and leukemic hematopoieses and transient clinical remission (33–35) due to the prolonged proliferative cycle of damaged HPCs (334–336). Along with an increase in leukemic cell burden due to dysregulated proliferative capacity (5–8), leukemic cells compromise the hematopoietic pool and outcompete their normal counterparts. Normal hematopoiesis is subsequently suppressed (337–340), which leads to the emergence of symptomatic MNs. The panorama of inflammatory stress-powered antileukemic immunity is better displayed in our reported patient with definitively diagnosed MDS with excessive blasts-1 who developed an aplastic crisis during an inflammatory episode (35). During the aplastic crisis, leukemic blasts regressed with atypical lymphocyte infiltration in the BM. Tentative antituberculosis treatment resulted in not only the disappearance of the atypical lymphocytes but also an increase in leukemic blasts.

### How should we interpret leukemic transformation during or shortly after ATG-based IST?

Approximately 70%–80% of SAA patients respond to ATG-based IST (19–23). However, dysregulation of the immune system and defects in HPCs persist even in remission patients (77, 78), and relapse eventually occurs in 30%–40% of responding patients (19–23), which indicates that the primary pathogenic factor is not removed by IST. In approximately 10%–15% of SAA patients, the disease phenotype transforms to MNs. In some cases, leukemic transformation occurs during or shortly after IST (19–23). Some MN-related somatic mutations in the SAA stage predict a lower frequency of deep hematologic response and a higher rate of disease relapse and leukemic transformation. Non-responding patients carry a higher frequency of these unfavorable mutations compared to responding patients (21–24). The presence of MN-related genetic aberrations influences treatment responses, which indicates that genetic damage in HPCs plays an important role in eliciting autoimmune pathogenesis. The pharmacologic role of ATG-based IST in AHF treatment is to deplete effector CTLs and induce immune tolerance; thus, IST functions as an immune suppressant to relieve autoimmune impairment in HPCs (28–32). At the same time, inflammatory stress-mediated antileukemic immunity can be compromised. After IST, the number and size of somatic mutations are massively increased (20, 42) due to the mitigation of inflammatory stress-powered antileukemic immunity. From this point of view, IST promotes the penetration of symptomatic MNs by interfering with the process of immunological attack, which is distinct from the removal of antigen stimulation by treating underlying infections. However, their effects are similar. Both IST and treatment of underlying infections can mitigate the intensity of the inflamed BME and promote the penetration of symptomatic MNs. Intriguingly, approximately one-third of patients who respond to IST do not relapse or progress to MNs, which supports the heterogeneous nature of the etiology and pathology that initiates and sustains autoimmune-mediated hematopoietic injuries. More interestingly, transient genetic aberrations occasionally occur in a paucity of patients after IST (19, 20). It is possible that some genetic aberrations are not correlated with or not sufficient for leukemic transformation (24, 25, 27).

## Particular attention should be given to gut inflammatory disorders and disseminated tuberculosis in AHF patients

Although patients with MNs may benefit from inflammatory stress-powered antileukemic immunity, an excessive inflammatory cascade and severe cytopenia can be harmful or even fatal. Like in other AIDs (156–161), AHF pathogenesis necessitates an active chronic inflammatory condition in addition to the inflamed BME to sustain BM-predominant autoimmune impairment (90, 95, 161, 313, 315). When AHF patients present with active inflammatory symptoms and their cytopenia is dramatically exacerbated, identifying and disposing of inflammatory niduses are urgent tasks that are needed to save patients’ lives. In this situation, particular attention should be given to inflammatory diseases in the gastrointestinal tract and disseminated tuberculosis. Tuberculosis reactivation may be the result of an immune exhausted state and/or the administration of immune suppressants. Disseminated tuberculosis can worsen hematopoietic function and become a major health problem in AHF patients.

### Association of gut inflammation with AHF pathogenesis

An association between AHF and gut inflammatory conditions has long been proposed (89, 90, 341). Patients with AA have a dysbiotic gut microbiota (342), and the immunological signature of AA is the same as that of IBD (343), which suggests that AA and IBD may share similar initiating factors and common pathogenic agents (1, 2, 344, 345). A gluten-free diet for coeliac disease-associated aplastic cytopenia (346) or resection of diseased colonic segments in patients with neutropenic enterocolitis (347) can effectively relieve hematopoietic suppression. In our previous report (348), two cycles of intermittent hematopoietic recovery and disease relapse occurred in a patient with refractory SAA following intermittent treatment with a gut-cleansing preparation, which reinforces the definitive role of gut inflammatory conditions in AHF pathogenesis. The contribution of the gut microbiome to sustaining the pathogenesis of graft-versus-host disease after allogeneic hematopoietic stem cell transplantation, a pathogenic mechanism similar to that of AA and frequently used to study AHF pathogenesis in animal models, was reported several decades ago (161). In our retrospective study, all patients with SAA had imaging abnormalities that reflected the existence of chronic inflammatory lesions and acutely aggravated inflammatory damage in the gastrointestinal tract during inflammatory episodes and exacerbated cytopenia (349). Similar to non-hematological AIDs (156–160), growing evidence demonstrates that the gastrointestinal tract is the most common inflammatory nidus that sustains AHF pathogenesis in patients with a background of an inflamed BME (89, 90). Aggravated gut inflammatory disorders can strengthen the immune-active BME and suppress normal and leukemic hematopoieses, which leads to AHF and regression of leukemic clones (33–36). The effective treatment of gut inflammatory conditions can ameliorate AHF (348) and promote leukemic transformation (33).

### Hematopoietic suppression by Th1 responses to tuberculosis

The Th1 immune response is the major mechanism involved in defense against *Mycobacterium tuberculosis*, with IFN-γ production playing a pivotal role in combating *M. tuberculosis* infection (291, 350, 351). IFN-γ is the most important mediator of AHF development (55, 56, 240) and links AHF pathogenesis with chronic infectious diseases that provoke Th1-predominant immune responses. Tuberculosis is one of the most common chronic intracellular infections (106, 209, 315). Over 90% of the world’s population has been infected by *M. tuberculosis*. Despite great therapeutic advances, nearly a quarter of individuals remain latently infected after the initial infection (352–354). Latent tuberculosis can be reactivated under certain circumstances such as with aging, undernourishment, prolonged psychological stress, long-term administration of immune suppressants, or coinfections with HIV or other pathogenic microbes (355–357). In this situation, antituberculosis immunity by trained Th1 cells (291, 358), CTLs (359, 360), NK/NKT cells (361, 362), unconventional lymphocytes (363, 364), and even CD5+ B cells (365) can be recalled. These memory immune cells rapidly expand and produce a substantial amount of IFN-γ, TNF-α, and other proinflammatory cytokines in response to increased *M. tuberculosis* antigen load. Excessive proinflammatory cytokines strengthen the primary immune-active BME and repress normal and leukemic hematopoieses (34, 35, 106, 315). Antigens of *M. tuberculosis* may also cross-react with autoantigens on HPCs. With the cross-reaction, reactivated effector immune cells induce an immunological attack on HPCs and strengthen the inflamed BME (88). Even in latent tuberculosis, chronic antigen stimulation may persist due to the high heterogeneity of this chronic infection (356, 365–367) and fluctuations in host performance status, which leads to fluctuating chronic inflammation.

### The more important role of gut involvement in tuberculosis infection

Disseminated tuberculosis (368–371) or even disseminated BCG vaccination (372) can induce BM hypoplasia and peripheral cytopenia. The fact that tuberculosis-induced AHF occurs only in disseminated tuberculosis instead of in isolated pulmonary tuberculosis suggests that tuberculosis can infect hematopoietic tissues (373) or the gastrointestinal tract (34, 35, 181, 349). Gut involvement and subsequent dysbiosis (181) likely play more important roles in inducing systemic inflammatory disorders (374, 375). In our retrospective study investigating infectious niduses during flared inflammatory episodes and accelerated cytopenia, five of 17 recruited SAA patients had imaging abnormalities suggestive of tuberculosis reactivation, all of which involved inflammatory diseases in the gastrointestinal tract (349). The incidence of tuberculosis reactivation may be even greater in advanced MN patients (376–378). It is not surprising that there is a high incidence of chronic *M. tuberculosis* infection in AHF patients because patients with HLA-DR15 have weaker responses to *M. tuberculosis* (379, 380), and AHF patients have a high frequency of HLA-DR15 phenotype (52, 53). Gastrointestinal and even isolated pulmonary tuberculosis can induce pathological processes in the gastrointestinal tract, not only alone but also in combination with gut dysbiosis, which results in diffuse gut inflammatory lesions (181, 381, 382). In this setting, both pathogenic and commensal (185, 186) microbes, in addition to *M. tuberculosis* infection (315, 316), become exogenous antigens to sustain deranged immune responses.

## Summary and perspective

Autoimmune responses in AHF may represent an inflammatory stress-powered antileukemic mechanism, at least in a significant proportion of patients whose pathogenesis is associated with MN-related genetic aberrations. With age, the incidence of clonal hematopoiesis increases, which is known as mutation-specific adaptation to environmental stressors and is consistent with a high incidence of AHF and inflammatory disorders in old age. However, age-related clonal hematopoiesis also increases the risk of malignant evolution (383–385). A dynamic balance between antileukemic activities and negative immune regulators may play a more important role in the suppression of transformed HPCs and the maintenance of effective hematopoiesis in old age. In this process, immune surveillance against neoplastic antigens or DAMPs initiates a primary inflammatory background in the BME and may contribute to the BM-specific autoimmune impairment. Extramedullary inflammatory stressors generated from exogenous antigen stimulation, especially from the gastrointestinal tract and chronic intracellular infections, play a critical role in the sustenance of autoimmune responses and the apoptosis of HPCs and promote the development of AA-like syndrome.

Although AA and MNs are generally considered distinctive disease entities, the phenotype of aplastic cytopenia can transform into MNs and vice versa. The switch that shapes the disease phenotype is a change in inflammation strength. During active infectious episodes, leukemic blasts can regress due to the reversion of the immune exhaustion state and the restoration of antileukemic activities. At this time, patients manifest aplastic cytopenia. After successful treatment of underlying infections, leukemic clones rapidly expand, and patients manifest symptomatic MNs, which commonly follow a short duration of hematological remission. Miscellaneous microbes or even endotoxins can induce antitumor immunity. This indicates that not antigen-specific stimulation but rather inflammatory stressors in response to microbial invasion are responsible for sustaining antitumor activities. Although they occur through different mechanisms, the effect of ATG-based IST is similar to that of treating underlying infections. Spontaneous remission in AML patients and non-specific immunological therapies in treating hematological malignancies may share an inflammatory stress-powered antileukemic mechanism.

An in-depth investigation of inflammatory stress-powered antileukemic immunity is highly important for theoretical research and clinical practice. Understanding the mechanisms of immune surveillance against malignant proliferation and the rational administration of immune-activating agents and ICIs in the treatment of hematological malignancies will be helpful, especially when aplastic cytopenia appears to be the major adverse effect. Some pathogenic microbes can stimulate Th1 responses. Inoculation of vaccines from these microbes has direct antileukemic effects and enhances ICI treatment efficacy. The inflammatory stress-powered antileukemic mechanism raises many questions.

1) Despite powering antileukemic immunity, chronic antigen stimulation may further exhaust the host immune system and upregulate the responsive threshold. These effects may lead to a requirement for more intensive antigen stimulation to achieve effective antileukemic strength, which forms a vicious cycle and attenuates antileukemic activities. This raises the question of how to take advantage of non-specific inoculation for the treatment of MNs without inducing immune exhaustion; intermittent inoculation in combination with ICI treatment is likely a superior option (348, 386, 387).
2) ICI treatments lower the response threshold, while inoculations enforce the response strength. ICI treatments in combination with inoculation may synergistically reinvigorate missed antileukemic activities and further enhance treatment efficacy. This combination therapy may produce an overwhelming inflammatory cascade and heavy hematopoietic suppression. This fatal side effect raises the question of how to tip the balance between benefits and severe toxicity and how to combine immunological therapies.
3) While some commensal microbes can stimulate Th1 responses and exhibit antileukemic activities, some microbes can suppress Th1 responses and favor the maintenance of an immune exhaustion state. However, the abundance and diversity of the gut microbiota are critical for the integrity and function of the gastrointestinal tract. Gut dysbiosis and excessive Th1 responses disrupt the intestinal integrity and barrier function and induce gut inflammatory conditions (147, 183, 197, 345). Furthermore, chronic gut inflammatory conditions can induce systemic immune dysregulation and immune exhaustion. This raises the question of how to exploit immune responses to “beneficial microbes” and avoid severe gut inflammatory damage.
4) Both AHF and MNs are fatal diseases. The resolution of inflammatory stressors by either IST or treatment of underlying diseases may lead to phenotypic transformation from AHF to clinically overt MNs, which complicates the patients’ disease course. This raises the question of how to assess the benefits and risks of leukemic transformation prior to IST. Additionally, underlying inflammatory conditions should also be properly managed.
5) Different lineages of immune cells are regulated by different cytokines in response to different antigen stimulation (307–310). Blood cells are immune cells, and MNs can be derived from different cell lineages (10). Some cytokines inhibit certain lineages of leukemic clones but may promote the proliferation of others (388). This raises the question of what leukemic cells of origin can benefit from and which ones may be promoted by the treatment with immune-activating agents.
6) It is unclear what antigens are neoplastic, what neoplastic antigens are immunogenic, and what intensity of immunogenic antigens can provoke antileukemic immunity. Although antileukemic immunity is powered by non-specific antigenic stimulation and immunogenic antigens responsible for antileukemic immunity may be miscellaneous in different neoplasms, is there any advantage in selecting an inoculation microbe with epitopes that cross-react with neoplastic antigens if the neoplastic antigens are identified?

Collectively, inflammatory stress-powered antileukemic immunity warrants extensive investigation. This may explore a new way of MN treatment. With in-depth investigations, many questions will emerge, and better strategies for improving treatment responses and managing adverse complications will be found.

## Author contributions

X-CZ: Conceptualization, Writing – original draft. BJ: Data curation, Writing – original draft. N-NX: Data curation, Writing – original draft. X-YS: Data curation, Writing – original draft. F-JM: Supervision, Writing – review & editing.

## References

[1] Giudice V Selleri C. Aplastic anemia: pathophysiology. Semin Hematol. (2022) 59:13–20. doi: 10.1053/j.seminhematol.2021.12.002 35491054

[2] Shallis RM Ahmad R Zeidan AM. Aplastic anemia: Etiology, molecular pathogenesis, and emerging concepts. Eur J Haematol. (2018) 101:711–20. doi: 10.1111/ejh.13153 30055055

[3] Killick SB Bown N Cavenagh J Dokal I Foukaneli T Hill A. Guidelines for the diagnosis and management of adult aplastic anaemia. Br J Haematol. (2016) 172:187–207. doi: 10.1111/bjh.13853 26568159

[4] Clucas DB Fox LC Wood EM Hong FS Gibson J Bajel A. Revisiting acquired aplastic anaemia: current concepts in diagnosis and management. Intern Med J. (2019) 49:152–9. doi: 10.1111/imj.14140 30324755

[5] Li H Hu F Gale RP Sekeres MA Liang Y. Myelodysplastic syndromes. Nat Rev Dis Primers. (2022) 8:74. doi: 10.1038/s41572-022-00402-5 36396662

[6] DiNardo CD Erba HP Freeman SD Wei AH. Acute myeloid leukaemia. Lancet. (2023) 401:2073–86. doi: 10.1016/S0140-6736(23)00108-3 37068505

[7] Singh H Kumar M Kanungo H. Role of gene mutations in acute myeloid leukemia: A review article. Glob Med Genet. (2023) 10:123–8. doi: 10.1055/s-0043-1770768 PMC10289861 37360004

[8] Bouligny IM Maher KR Grant S. Mechanisms of myeloid leukemogenesis: Current perspectives and therapeutic objectives. Blood Rev. (2022) 2022:100996. doi: 10.1016/j.blre.2022.100996 PMC10693933 35989139

[9] Baidurin S Akhmetzhanova S Ilmalieva A Sagyndykova G Orazbekova A. Myelodysplastic syndrome: diagnosis, treatment and prognosis (LITERATURE REVIEW). Georgian Med News. (2023) 334:103–7. 36864802

[10] Khoury JD Solary E Abla O Akkari Y Alaggio R Apperley JF. The 5th edition of the world health organization classification of haematolymphoid tumours: myeloid and histiocytic/dendritic neoplasms. Leukemia. (2022) 36:1703–19. doi: 10.1038/s41375-022-01613-1 PMC9252913 35732831

[11] Mufti GJ Hamblin TJ Lee-Potter JP. The aplasia-leukaemia syndrome. Aplastic anaemia followed by dyserythropoiesis, myeloproliferative syndrome and acute myeloid leukaemia. Acta Haematol. (1983) 69:349–52. doi: 10.1159/000206918 6404118

[12] Sun L Babushok DV. Secondary myelodysplastic syndrome and leukemia in acquired aplastic anemia and paroxysmal nocturnal hemoglobinuria. Blood. (2020) 136:36–49. doi: 10.1182/blood.2019000940 32430502 PMC7332901

[13] de Planque MM Kluin-Nelemans HC van Krieken HJ Kluin PM Brand A Beverstock GC. Evolution of acquired severe aplastic anaemia to myelodysplasia and subsequent leukaemia in adults. Br J Haematol. (1988) 70:55–62. doi: 10.1111/j.1365-2141.1988.tb02434.x 3179229

[14] Ohara A Kojima S Hamajima N Tsuchida M Imashuku S Ohta S. Myelodysplastic syndrome and acute myelogenous leukemia as a late clonal complication in children with acquired aplastic anemia. Blood. (1997) 90:1009–13. 9242530

[15] Zambetti NA Ping Z Chen S Kenswil KJG Mylona MA Sanders MA. Mesenchymal inflammation drives genotoxic stress in hematopoietic stem cells and predicts disease evolution in human pre-leukemia. Cell Stem Cell. (2016) 19:613–27. doi: 10.1016/j.stem.2016.08.021 27666011

[16] Burocziova M Grusanovic S Vanickova K Kosanovic S Alberich-Jorda M. Chronic inflammation promotes cancer progression as a second hit. Exp Hematol. (2023) 13:S0301–472X(23)01702-2. doi: 10.1016/j.exphem.2023.09.002 37709251

[17] Gurnari C Pagliuca S Maciejewski JP. Clonal evolution in aplastic anemia: failed tumor surveillance or maladaptive recovery? Leuk Lymphoma. (2023) 64:1389–99. doi: 10.1080/10428194.2023.2215614 PMC11104022 37356012

[18] Durrani J Groarke EM. Clonality in immune aplastic anemia: Mechanisms of immune escape or Malignant transformation. Semin Hematol. (2022) 59:137–42. doi: 10.1053/j.seminhematol.2022.08.001 PMC9938528 36115690

[19] Scheinberg P. Recent advances and long-term results of medical treatment of acquired aplastic anemia: are patients cured? Hematol Oncol Clin North Am. (2018) 32:609–18. doi: 10.1016/j.hoc.2018.03.003 30047414

[20] Peffault de Latour R Kulasekararaj A Iacobelli S Terwel SR Cook R Griffin M. Eltrombopag added to immunosuppression in severe aplastic anemia. N Engl J Med. (2022) 386:11–23. doi: 10.1056/NEJMoa2109965 34986284

[21] Groarke EM Patel BA Shalhoub R Gutierrez-Rodrigues F Desai P Leuva H. Predictors of clonal evolution and myeloid neoplasia following immunosuppressive therapy in severe aplastic anemia. Leukemia. (2022) 36:2328–37. doi: 10.1038/s41375-022-01636-8 PMC9701554 35896822

[22] Kulasekararaj AG Jiang J Smith AE Mohamedali AM Mian S Gandhi S. Somatic mutations identify a subgroup of aplastic anemia patients who progress to myelodysplastic syndrome. Blood. (2014) 124:2698–704. doi: 10.1182/blood-2014-05-574889 PMC4383793 25139356

[23] Kojima S Ohara A Tsuchida M Kudoh T Hanada R Okimoto Y. Risk factors for evolution of acquired aplastic anemia into myelodysplastic syndrome and acute myeloid leukemia after immunosuppressive therapy in children. Blood. (2002) 100:786–90. doi: 10.1182/blood.v100.3.786 12130487

[24] Ogawa S. MDS-related mutations in aplastic anemia. Blood. (2014) 124:2619–20. doi: 10.1182/blood-2014-09-597708 25342663

[25] Liu L Zhang D Fu Q Wang J Yu J Chen D. Clinical implications of myeloid Malignancy-related somatic mutations in aplastic anemia. Clin Exp Med. (2023) 23:4473–82. doi: 10.1007/s10238-023-01067-4 PMC10725342 37087521

[26] Patel BA Ghannam J Groarke EM Goswami M Dillon L Gutierrez-Rodrigues F. Detectable mutations precede late myeloid neoplasia in aplastic anemia. Haematologica. (2021) 106:647–50. doi: 10.3324/haematol.2020.263046 PMC7849750 33054127

[27] Brzeźniakiewicz-Janus K Rupa-Matysek J Gil L. Acquired aplastic anemia as a clonal disorder of hematopoietic stem cells. Stem Cell Rev Rep. (2020) 16:472–81. doi: 10.1007/s12015-020-09971-y PMC7253510 32270433

[28] Mohty M. Mechanisms of action of antithymocyte globulin: T-cell depletion and beyond. Leukemia. (2007) 21:1387–94. doi: 10.1038/sj.leu.2404683 17410187

[29] Duftner C Dejaco C Hengster P Bijuklic K Joannidis M Margreiter R. Apoptotic effects of antilymphocyte globulins on human pro-inflammatory CD4+CD28- T-cells. PloS One. (2012) 7:e33939. doi: 10.1371/journal.pone.0033939 22479483 PMC3316508

[30] Dubey S Srivastava A Nityanand S. Induction of apoptosis of peripheral blood mononuclear cells by antithymocyte globulin (ATG) in aplastic anemia: an in *vivo* and in *vitro* study. Ann Hematol. (2002) 81:249–53. doi: 10.1007/s00277-002-0454-7 12029533

[31] Liu Z Fang Y Wang X Wang P Yun P Xu H. Upregulation of molecules associated with T-regulatory function by thymoglobulin pretreatment of human CD4+ cells. Transplantation. (2008) 86:1419–26. doi: 10.1097/TP.0b013e318187c2e5 19034013

[32] Copic D Direder M Klas K Bormann D Laggner M Ankersmit HJ. Antithymocyte globulin inhibits CD8+ T cell effector functions via the paracrine induction of PDL-1 on monocytes. Cells. (2023) 12:382. doi: 10.3390/cells12030382 36766722 PMC9913606

[33] Zhao XC Sun XY Ju B Meng FJ Zhao HG. Acquired aplastic anemia: Is bystander insult to autologous hematopoiesis driven by immune surveillance against Malignant cells? World J Stem Cells. (2020) 12:1429–38. doi: 10.4252/wjsc.v12.i11.1429 PMC7705466 33312408

[34] Xiu NN Yang XD Xu J Ju B Sun XY Zhao XC. Leukemic transformation during anti-tuberculosis treatment in aplastic anemia-paroxysmal nocturnal hemoglobinuria syndrome: A case report and review of literature. World J Clin cases. (2023) 11:6908–19. doi: 10.12998/wjcc.v11.i28.6908 PMC10600849 37901004

[35] Sun XY Yang XD Xu J Xiu NN Ju B Zhao XC. Tuberculosis-induced aplastic crisis and atypical lymphocyte expansion in advanced myelodysplastic syndrome: A case report and review of literature. World J Clin cases. (2023) 11:4713–22. doi: 10.12998/wjcc.v11.i19.4713 PMC10353497 37469724

[36] Ju B Xiu NN Xu J Yang XD Sun XY Zhao XC. Flared inflammatory episode transforms advanced myelodysplastic syndrome into aplastic pancytopenia: A case report and literature review. World J Clin cases. (2023) 11:4105–16. doi: 10.12998/wjcc.v11.i17.4105 PMC10303598 37388797

[37] Skibenes ST Clausen I Raaschou-Jensen K. Next-generation sequencing in hypoplastic bone marrow failure: What difference does it make? Eur J Haematol. (2021) 106:3–13. doi: 10.1111/ejh.13513 32888355

[38] Zheng G Chen P Pallavajjalla A Haley L Gondek L Dezern A. The diagnostic utility of targeted gene panel sequencing in discriminating etiologies of cytopenia. Am J Hematol. (2019) 94:1141–8. doi: 10.1002/ajh.25592 PMC9162094 31350794

[39] Votavova H Belickova M. Hypoplastic myelodysplastic syndrome and acquired aplastic anemia: Immune-mediated bone marrow failure syndromes (Review). Int J Oncol. (2022) 60:7. doi: 10.3892/ijo.2021.5297 34958107 PMC8727136

[40] Giudice V Cardamone C Triggiani M Selleri C. Bone marrow failure syndromes, overlapping diseases with a common cytokine signature. Int J Mol Sci. (2021) 22:705. doi: 10.3390/ijms22020705 33445786 PMC7828244

[41] Li X Xu F He Q Wu L Zhang Z Chang C. Comparison of immunological abnormalities of lymphocytes in bone marrow in myelodysplastic syndrome (MDS) and aplastic anemia (AA). Intern Med. (2010) 49:1349–55. doi: 10.2169/internalmedicine.49.3477 20647647

[42] Fattizzo B Levati GV Giannotta JA Cassanello G Cro LM Zaninoni A. Low-risk myelodysplastic syndrome revisited: morphological, autoimmune, and molecular features as predictors of outcome in a single center experience. Front Oncol. (2022) 12:795955. doi: 10.3389/fonc.2022.795955 35392224 PMC8980524

[43] Kook H Zeng W Guibin C Kirby M Young NS Maciejewski JP. Increased cytotoxic T cells with effector phenotype in aplastic anemia and myelodysplasia. Exp Hematol. (2001) 29:1270–7. doi: 10.1016/s0301-472x(01)00736-6 11698122

[44] You X Yang Q Yan K Wang SR Huang RR Wang SQ. Multi-omics profiling identifies pathways associated with CD8+ T-cell activation in severe aplastic anemia. Front Genet. (2022) 12:790990. doi: 10.3389/fgene.2021.790990 35058969 PMC8764265

[45] Gravano DM Al-Kuhlani M Davini D Sanders PD Manilay JO Hoyer KK. CD8+ T cells drive autoimmune hematopoietic stem cell dysfunction and bone marrow failure. J Autoimmun. (2016) 75:58–67. doi: 10.1016/j.jaut.2016.07.007 27453063 PMC5121063

[46] Liu C Li Z Sheng W Fu R Li L Zhang T. Abnormalities of quantities and functions of natural killer cells in severe aplastic anemia. Immunol Invest. (2014) 43:491–503. doi: 10.3109/08820139.2014.888448 24661133

[47] Chen T Liu C Li L Liu H Wang T Shao Z. CD56^bright^ natural killer cells exhibit abnormal phenotype and function in severe aplastic anemia. Int J Lab Hematol. (2019) 41:353–63. doi: 10.1111/ijlh.12982 30779419

[48] Yu H Liu H Zhao Y Wang H Liu C Qi W. Upregulated expression of leukocyte immunoglobulin-like receptor A3 in patients with severe aplastic anemia. Exp Ther Med. (2021) 21:346. doi: 10.3892/etm.2021.9777 33732319 PMC7903422

[49] Patel BA Giudice V Young NS. Immunologic effects on the haematopoietic stem cell in marrow failure. Best Pract Res Clin Haematol. (2021) 34:101276. doi: 10.1016/j.beha.2021.101276 34404528

[50] Giudice V Feng X Lin Z Hu W Zhang F Qiao W. Deep sequencing and flow cytometric characterization of expanded effector memory CD8+CD57+ T cells frequently reveals T-cell receptor Vβ oligoclonality and CDR3 homology in acquired aplastic anemia. Haematologica. (2018) 103:759–69. doi: 10.3324/haematol.2017.176701 PMC5927970 29419434

[51] Dufour C Corcione A Svahn J Haupt R Battilana N Pistoia V. Interferon gamma and tumour necrosis factor alpha are overexpressed in bone marrow T lymphocytes from paediatric patients with aplastic anaemia. Br J Haematol. (2001) 115:1023–31. doi: 10.1046/j.1365-2141.2001.03212.x 11843845

[52] Saunthararajah Y Nakamura R Nam JM Robyn J Loberiza F Maciejewski JP. HLA-DR15 (DR2) is overrepresented in myelodysplastic syndrome and aplastic anemia and predicts a response to immunosuppression in myelodysplastic syndrome. Blood. (2002) 100:1570–4. doi: 10.1182/blood.V100.5.1570.h81702001570_1570_1574 12176872

[53] Sugimori C Yamazaki H Feng X Mochizuki K Kondo Y Takami A. Roles of DRB1 *1501 and DRB1 *1502 in the pathogenesis of aplastic anemia. Exp Hematol. (2007) 35:13–20. doi: 10.1016/j.exphem.2006.09.002 17198869

[54] Liu H Mihara K Kimura A Tanaka K Kamada N. Induction of apoptosis in CD34+ cells by sera from patients with aplastic anemia. Hiroshima J Med Sci. (1999) 48:57–63. 10434475

[55] Chen J Feng X Desierto MJ Keyvanfar K Young NS. IFN-γ-mediated hematopoietic cell destruction in murine models of immune-mediated bone marrow failure. Blood. (2015) 126:2621–31. doi: 10.1182/blood-2015-06-652453 PMC4671109 26491068

[56] Qin Y Fang K Lu N Hu Y Tian Z Zhang C. Interferon gamma inhibits the differentiation of mouse adult liver and bone marrow hematopoietic stem cells by inhibiting the activation of notch signaling. Stem Cell Res Ther. (2019) 10:210. doi: 10.1186/s13287-019-1311-0 31311586 PMC6636148

[57] Chen Y Zou Z Wu Z Zhao Z Luo X Xie C. TNF-α-induced programmed cell death in the pathogenesis of acquired aplastic anemia. Expert Rev Hematol. (2015) 8:515–26. doi: 10.1586/17474086.2015.1049593 26149913

[58] Balakrishnan A Jama B Morris GP. Endogenous co-expression of two T cell receptors promotes lymphopenia-induced proliferation via increased affinity for self-antigen. J Leukoc Biol. (2018) 104:1097–104. doi: 10.1002/JLB.1AB0618-214RRR 30168881

[59] Nakao S Takamatsu H Yachie A Itoh T Yamaguchi M Ueda M. Establishment of a CD4+ T cell clone recognizing autologous hematopoietic progenitor cells from a patient with immune-mediated aplastic anemia. Exp Hematol. (1995) 23:433–8. 7720814

[60] Tang Y Desierto MJ Chen J Young NS. The role of the Th1 transcription factor T-bet in a mouse model of immune-mediated bone-marrow failure. Blood. (2010) 115:541–8. doi: 10.1182/blood-2009-03-211383 PMC2810980 19903901

[61] Maciejewski JP Selleri C Sato T Anderson S Young NS. Increased expression of Fas antigen on bone marrow CD34+ cells of patients with aplastic anaemia. Br J Haematol. (1995) 91:245–52. doi: 10.1111/j.1365-2141.1995.tb05277.x 7577642

[62] Omokaro SO Desierto MJ Eckhaus MA Ellison FM Chen J Young NS. Lymphocytes with aberrant expression of Fas or Fas ligand attenuate immune bone marrow failure in a mouse model. J Immunol. (2009) 182:3414–22. doi: 10.4049/jimmunol.0801430 PMC3513944 19265119

[63] Kakagianni T Giannakoulas NC Thanopoulou E Galani A Michalopoulou S Kouraklis-Symeonidis A. A probable role for trail-induced apoptosis in the pathogenesis of marrow failure. Implications from an in *vitro* model and from marrow of aplastic anemia patients. Leuk Res. (2006) 30:713–21. doi: 10.1016/j.leukres.2005.09.015 16310248

[64] Ball SE Gibson FM Rizzo S Tooze JA Marsh JC Gordon-Smith EC. Progressive telomere shortening in aplastic anemia. Blood. (1998) 91:3582–92. 9572992

[65] Bär C Povedano JM Serrano R Benitez-Buelga C Popkes M Formentini I. Telomerase gene therapy rescues telomere length, bone marrow aplasia, and survival in mice with aplastic anemia. Blood. (2016) 127:1770–9. doi: 10.1182/blood-2015-08-667485 26903545

[66] Maratheftis CI Andreakos E Moutsopoulos HM Voulgarelis M. Toll-like receptor-4 is up-regulated in hematopoietic progenitor cells and contributes to increased apoptosis in myelodysplastic syndromes. Clin Cancer Res. (2007) 13:1154–60. doi: 10.1158/1078-0432.CCR-06-2108 17317824

[67] Hsu HC Tsai WH Chen LY Hsu ML Ho CH Lin CK. Overproduction of inhibitory hematopoietic cytokines by lipopolysaccharide-activated peripheral blood mononuclear cells in patients with aplastic anemia. Ann Hematol. (1995) 71:281–6. doi: 10.1007/BF01697980 8534759

[68] Banerjee T Calvi LM Becker MW Liesveld JL. Flaming and fanning: The Spectrum of inflammatory influences in myelodysplastic syndromes. Blood Rev. (2019) 36:57–69. doi: 10.1016/j.blre.2019.04.004 31036385 PMC6711159

[69] Paracatu LC Schuettpelz LG. Contribution of aberrant toll like receptor signaling to the pathogenesis of myelodysplastic syndromes. Front Immunol. (2020) 11:1236. doi: 10.3389/fimmu.2020.01236 32625214 PMC7313547

[70] Yan L Fu R Liu H Wang H Liu C Wang T. Abnormal quantity and function of regulatory T cells in peripheral blood of patients with severe aplastic anemia. Cell Immunol. (2015) 296:95–105. doi: 10.1016/j.cellimm.2015.04.001 25906694

[71] Kordasti S Costantini B Seidl T Perez Abellan P Martinez Llordella M McLornan D. Deep phenotyping of Tregs identifies an immune signature for idiopathic aplastic anemia and predicts response to treatment. Blood. (2016) 128:1193–205. doi: 10.1182/blood-2016-03-703702 PMC5009512 27281795

[72] Fang J Lin L Wang Y Lin D Liu C Sunlong Q. Regulatory T cells and CD20+ B cells in pediatric very severe aplastic anemia: possible clinical markers for evaluating the therapeutic efficacy and prognosis. Hematology. (2018) 23:823–7. doi: 10.1080/10245332.2018.1498566 29996743

[73] Zaimoku Y Patel BA Kajigaya S Feng X Alemu L Quinones Raffo D. Deficit of circulating CD19+CD24hiCD38hi regulatory B cells in severe aplastic anaemia. Br J Haematol. (2020) 190:610–7. doi: 10.1111/bjh.16651 PMC7496711 32311088

[74] Taketazu F Miyagawa K Ichijo H Oshimi K Mizoguchi H Hirai H. Decreased level of transforming growth factor-beta in blood lymphocytes of patients with aplastic anemia. Growth Factors. (1992) 6:85–90. doi: 10.3109/08977199209008874 1591018

[75] Asano Y Shibata S Kobayashi S Okamura S Niho Y. Effect of interleukin 10 on the hematopoietic progenitor cells from patients with aplastic anemia. Stem Cells. (1999) 17:147–51. doi: 10.1002/stem.170147 10342557

[76] Yu W Ge M Lu S Shi J Li X Zhang J. Anti-inflammatory effects of interleukin-35 in acquired aplastic anemia. Cytokine. (2015) 76:409–16. doi: 10.1016/j.cyto.2015.08.004 26282938

[77] Maciejewski JP Kim S Sloand E Selleri C Young NS. Sustained long-term hematologic recovery despite a marked quantitative defect in the stem cell compartment of patients with aplastic anemia after immunosuppressive therapy. Am J Hematol. (2000) 65:123–31. doi: 10.1002/1096-8652(200010)65:2<123::aid-ajh6>3.0.co;2-m 10996829

[78] Giannakoulas NC Karakantza M Theodorou GL Pagoni M Galanopoulos A Kakagianni T. Clinical relevance of balance between type 1 and type 2 immune responses of lymphocyte subpopulations in aplastic anaemia patients. Br J Haematol. (2004) 124:97–105. doi: 10.1046/j.1365-2141.2003.04729.x 14675414

[79] de Planque MM van Krieken JH Kluin-Nelemans HC Colla LP van der Burgh F Brand A. Bone marrow histopathology of patients with severe aplastic anaemia before treatment and at follow-up. Br J Haematol. (1989) 72:439–44. doi: 10.1111/j.1365-2141.1989.tb07729.x 2788455

[80] Zeng W Kajigaya S Chen G Risitano AM Nunez O Young NS. Transcript profile of CD4+ and CD8+ T cells from the bone marrow of acquired aplastic anemia patients. Exp Hematol. (2004) 32:806–14. doi: 10.1016/j.exphem.2004.06.004 15345281

[81] Melenhorst JJ van Krieken JH Dreef E Landegent JE Willemze R Fibbe WE. T cells selectively infiltrate bone marrow areas with residual haemopoiesis of patients with acquired aplastic anaemia. Br J Haematol. (1997) 99:517–9. doi: 10.1046/j.1365-2141.1997.4353245.x 9401058

[82] Selleri C Maciejewski JP Sato T Young NS. Interferon-gamma constitutively expressed in the stromal microenvironment of human marrow cultures mediates potent hematopoietic inhibition. Blood. (1996) 87:4149–57. 8639773

[83] Liu B Shao Y Liu Z Liu C Zhang T Fu R. Bone marrow plasma cytokine signature profiles in severe aplastic anemia. BioMed Res Int. (2020) 2020:8789275. doi: 10.1155/2020/8789275 32190686 PMC7063880

[84] Behzadi Fard M Kaviani S Atashi A. Parvovirus B19 infection in human bone marrow mesenchymal stem cells affects gene expression of IL-6 and TNF-α and also affects hematopoietic stem cells differentiation. Indian J Hematol Blood Transfus. (2019) 35:765–72. doi: 10.1007/s12288-019-01097-7 PMC6825089 31741634

[85] Zhang T Liu C Liu H Li L Wang T Fu R. Epstein barr virus infection affects function of cytotoxic T lymphocytes in patients with severe aplastic anemia. BioMed Res Int. (2018) 2018:6413815. doi: 10.1155/2018/6413815 29862282 PMC5976969

[86] Forte E Zhang Z Thorp EB Hummel M. Cytomegalovirus latency and reactivation: an intricate interplay with the host immune response. Front Cell Infect Microbiol. (2020) 10:130. doi: 10.3389/fcimb.2020.00130 32296651 PMC7136410

[87] Johns JL Macnamara KC Walker NJ Winslow GM Borjesson DL. Infection with Anaplasma phagocytophilum induces multilineage alterations in hematopoietic progenitor cells and peripheral blood cells. Infect Immun. (2009) 77:4070–80. doi: 10.1128/IAI.00570-09 PMC2738004 19564373

[88] Giudice V Risitano AM Selleri C. Infectious agents and bone marrow failure: A causal or a casual connection? Front Med (Lausanne). (2021) 8:757730. doi: 10.3389/fmed.2021.757730 34805223 PMC8599277

[89] Espinoza JL Elbadry MI Nakao S. An altered gut microbiota may trigger autoimmune-mediated acquired bone marrow failure syndromes. Clin Immunol. (2016) 171:62–4. doi: 10.1016/j.clim.2016.08.008 27506961

[90] Zhao XC Sun XY Zhao L Meng FJ. Gut inflammation in the pathogenesis of acquired aplastic anemia. Chin Med J (Engl). (2020) 133:1878–81. doi: 10.1097/CM9.0000000000000772 PMC7469989 32568881

[91] Yu S Sun Y Shao X Zhou Y Yu Y Kuai X. Leaky gut in IBD: intestinal barrier-gut microbiota interaction. J Microbiol Biotechnol. (2022) 32:825–34. doi: 10.4014/jmb.2203.03022 PMC9628915 35791076

[92] Mu Q Kirby J Reilly CM Luo XM. Leaky gut as a danger signal for autoimmune diseases. Front Immunol. (2017) 8:598. doi: 10.3389/fimmu.2017.00598 28588585 PMC5440529

[93] Xie X Su M Ren K Ma X Lv Z Li Z. Clonal hematopoiesis and bone marrow inflammation. Transl Res. (2023) 255:159–70. doi: 10.1016/j.trsl.2022.11.004 PMC11992924 36347490

[94] Ahmad MH Hegde M Wong WJ M Hosseini M Garrett LJ Carrascoso A. Runx1-R188Q germline mutation induces inflammation and predisposition to hematologic Malignancies in mice. Blood Adv. (2023) 27:2023010398. doi: 10.1182/bloodadvances.2023010398 PMC10711191 37756546

[95] Xin J Breslin P Wei W Li J Gutierrez R Cannova J. Necroptosis in spontaneously-mutated hematopoietic cells induces autoimmune bone marrow failure in mice. Haematologica. (2017) 102:295–307. doi: 10.3324/haematol.2016.151514 27634200 PMC5286937

[96] Biavasco R Lettera E Giannetti K Gilioli D Beretta S Conti A. Oncogene-induced senescence in hematopoietic progenitors features myeloid restricted hematopoiesis, chronic inflammation and histiocytosis. Nat Commun. (2021) 12:4559. doi: 10.1038/s41467-021-24876-1 34315896 PMC8316479

[97] Monlish DA Bhatt ST Duncavage EJ Greenberg ZJ Keller JL Romine MP. Loss of Toll-like receptor 2 results in accelerated leukemogenesis in the NUP98-HOXD13 mouse model of MDS. Blood. (2018) 131:1032–35. doi: 10.1182/blood-2017-08-801944 PMC5833264 29358180

[98] Ratajczak MZ Bujko K Cymer M Thapa A Adamiak M Ratajczak J. The Nlrp3 inflammasome as a “rising star” in studies of normal and Malignant hematopoiesis. Leukemia. (2020) 34:1512–23. doi: 10.1038/s41375-020-0827-8 PMC7266743 32313108

[99] Fraison JB Grignano E Braun T Adès L Chollet-Martin S Roland-Nicaise P. Autoantibodies in myelodysplastic syndromes and chronic myelomonocytic leukemia. Leuk Lymphoma. (2019) 60:2594–96. doi: 10.1080/10428194.2019.1599114 30958078

[100] Forghieri F Riva G Lagreca I Barozzi P Bettelli F Paolini A. Neoantigen-specific T-cell immune responses: the paradigm of NPM1-mutated acute myeloid leukemia. Int J Mol Sci. (2021) 22:9159. doi: 10.3390/ijms22179159 34502069 PMC8431540

[101] Menteşe A Erkut N Demir S Özer Yaman S Sümer A Doğramacı Ş. Autoantibodies against carbonic anhydrase I and II in patients with acute myeloid leukemia. Turk J Haematol. (2017) 34:307–13. doi: 10.4274/tjh.2016.0341 PMC5774362 28270370

[102] Jankovicova B Skultety L Dubrovcakova M Stern M Bilkova Z Lakota J. Overlap of epitopes recognized by anti-carbonic anhydrase I IgG in patients with Malignancy-related aplastic anemia-like syndrome and in patients with aplastic anemia. Immunol Lett. (2013) 153:47–9. doi: 10.1016/j.imlet.2013.07.006 23892086

[103] Jara LJ Medina G Saavedra MA. Autoimmune manifestations of infections. Curr Opin Rheumatol. (2018) 30:373–9. doi: 10.1097/BOR.0000000000000505 29528865

[104] Johnson D Jiang W. Infectious diseases, autoantibodies, and autoimmunity. J Autoimmun. (2022) 137:102962. doi: 10.1016/j.jaut.2022.102962 36470769 PMC10235211

[105] Olson JK Croxford JL Miller SD. Virus-induced autoimmunity: potential role of viruses in initiation, perpetuation, and progression of T-cell-mediated autoimmune disease. Viral Immunol. (2001) 14:227–50. doi: 10.1089/088282401753266756 11572634

[106] Belyaeva IV Kosova AN Vasiliev AG. Tuberculosis and autoimmunity. Pathophysiology. (2022) 29:298–318. doi: 10.3390/pathophysiology29020022 35736650 PMC9228380

[107] Wang L Cao ZM Zhang LL Dai XC Liu ZJ Zeng YX. Helicobacter pylori and autoimmune diseases: involving multiple systems. Front Immunol. (2022) 13:833424. doi: 10.3389/fimmu.2022.833424 35222423 PMC8866759

[108] Flores-Chávez A Carrion JA Forns X Ramos-Casals M. Extrahepatic manifestations associated with Chronic Hepatitis C Virus Infection. Rev Esp Sanid Penit. (2017) 19:87–97. doi: 10.4321/S1575-06202017000300004 29364334 PMC6241927

[109] Houen G Trier NH. Epstein-barr virus and systemic autoimmune diseases. Front Immunol. (2021) 11:587380. doi: 10.3389/fimmu.2020.587380 33488588 PMC7817975

[110] Al-Beltagi M Saeed NK Bediwy AS. COVID-19 disease and autoimmune disorders: A mutual pathway. World J Methodol. (2022) 12:200–23. doi: 10.5662/wjm.v12.i4.200 PMC9350728 36159097

[111] Torres-Aguilar H Sosa-Luis SA Aguilar-Ruiz SR. Infections as triggers of flares in systemic autoimmune diseases: novel innate immunity mechanisms. Curr Opin Rheumatol. (2019) 31:525–31. doi: 10.1097/BOR.0000000000000630 31135383

[112] Bao Y Dong C Ji J Gu Z. Dysregulation of gut microbiome is linked to disease activity of rheumatic diseases. Clin Rheumatol. (2020) 39:2523–8. doi: 10.1007/s10067-020-05170-9 32519049

[113] Yu W Jiang N Ebert PJ Kidd BA Müller S Lund PJ. Clonal deletion prunes but does not eliminate self-specific αβ CD8(+) T lymphocytes. Immunity. (2015) 42:929–41. doi: 10.1016/j.immuni.2015.05.001 PMC4455602 25992863

[114] Ni PP Solomon B Hsieh CS Allen PM Morris GP. The ability to rearrange dual TCRs enhances positive selection, leading to increased Allo- and Autoreactive T cell repertoires. J Immunol. (2014) 193:1778–86. doi: 10.4049/jimmunol.1400532 PMC4119549 25015825

[115] Lee DS Ahn C Ernst B Sprent J Surh CD. Thymic selection by a single MHC/peptide ligand: autoreactive T cells are low-affinity cells. Immunity. (1999) 10:83–92. doi: 10.1016/s1074-7613(00)80009-6 10023773

[116] Yan J Mamula MJ. B and T cell tolerance and autoimmunity in autoantibody transgenic mice. Int Immunol. (2002) 14:963–71. doi: 10.1093/intimm/dxf064 12147633

[117] Beignon AS Galeotti C Menager MM Schvartz A. Trained immunity as a possible newcomer in autoinflammatory and autoimmune diseases pathophysiology. Front Med (Lausanne). (2023) 9:1085339. doi: 10.3389/fmed.2022.1085339 36743677 PMC9896524

[118] Mora VP Loaiza RA Soto JA Bohmwald K Kalergis AM. Involvement of trained immunity during autoimmune responses. J Autoimmun. (2022) 14:102956. doi: 10.1016/j.jaut.2022.102956 36526524

[119] Municio C Criado G. Therapies targeting trained immune cells in inflammatory and autoimmune diseases. Front Immunol. (2021) 11:631743. doi: 10.3389/fimmu.2020.631743 33569065 PMC7868395

[120] Rojas M Restrepo-Jiménez P Monsalve DM Pacheco Y Acosta-Ampudia Y Ramírez-Santana C. Molecular mimicry and autoimmunity. J Autoimmun. (2018) 95:100–23. doi: 10.1016/j.jaut.2018.10.012 30509385

[121] Garabatos N Santamaria P. Gut microbial antigenic mimicry in autoimmunity. Front Immunol. (2022) 13:873607. doi: 10.3389/fimmu.2022.873607 35572569 PMC9094498

[122] Segal Y Shoenfeld Y. Vaccine-induced autoimmunity: the role of molecular mimicry and immune crossreaction. Cell Mol Immunol. (2018) 15:586–94. doi: 10.1038/cmi.2017.151 PMC6078966 29503439

[123] Begum S Aiman S Ahmad S Samad A Almehmadi M Allahyani M. Molecular mimicry analyses unveiled the human herpes simplex and poxvirus epitopes as possible candidates to incite autoimmunity. Pathogens. (2022) 11:1362. doi: 10.3390/pathogens11111362 36422613 PMC9696880

[124] Pacheco Y Acosta-Ampudia Y Monsalve DM Chang C Gershwin ME Anaya JM. Bystander activation and autoimmunity. J Autoimmun. (2019) 103:102301. doi: 10.1016/j.jaut.2019.06.012 31326230

[125] Lee HG Cho MZ Choi JM. Bystander CD4+ T cells: crossroads between innate and adaptive immunity. Exp Mol Med. (2020) 52:1255–63. doi: 10.1038/s12276-020-00486-7 PMC8080565 32859954

[126] Shim CH Cho S Shin YM Choi JM. Emerging role of bystander T cell activation in autoimmune diseases. BMB Rep. (2022) 55:57–64. doi: 10.5483/BMBRep.2022.55.2.183 35000675 PMC8891623

[127] Vanderlugt CL Miller SD. Epitope spreading in immune-mediated diseases: implications for immunotherapy. Nat Rev Immunol. (2002) 2:85–95. doi: 10.1038/nri724 11910899

[128] Cornaby C Gibbons L Mayhew V Sloan CS Welling A Poole BD. B cell epitope spreading: mechanisms and contribution to autoimmune diseases. Immunol Lett. (2015) 163:56–68. doi: 10.1016/j.imlet.2014.11.001 25445494

[129] Maverakis E Kim K Shimoda M Gershwin ME Patel F Wilken R. Glycans in the immune system and The Altered Glycan Theory of Autoimmunity: a critical review. J Autoimmun. (2015) 57:1–13. doi: 10.1016/j.jaut.2014.12.002 25578468 PMC4340844

[130] Epp A Sullivan KC Herr AB Strait RT. Immunoglobulin glycosylation effects in allergy and immunity. Curr Allergy Asthma Rep. (2016) 16:79. doi: 10.1007/s11882-016-0658-x 27796794

[131] Maldonado RF Sá-Correia I Valvano MA. Lipopolysaccharide modification in Gram-negative bacteria during chronic infection. FEMS Microbiol Rev. (2016) 40:480–93. doi: 10.1093/femsre/fuw007 PMC4931227 27075488

[132] Hsu YP Frank M Mukherjee D Shchurik V Makarov A Mann BF. Structural remodeling of SARS-CoV-2 spike protein glycans reveals the regulatory roles in receptor-binding affinity. Glycobiology. (2023) 33:126–37. doi: 10.1093/glycob/cwac077 PMC9990995 36370046

[133] Noli Truant S Redolfi DM Sarratea MB Malchiodi EL Fernández MM. Superantigens, a paradox of the immune response. Toxins (Basel). (2022) 14:800. doi: 10.3390/toxins14110800 36422975 PMC9692936

[134] Deacy AM Gan SK Derrick JP. Superantigen recognition and interactions: functions, mechanisms and applications. Front Immunol. (2021) 12:731845. doi: 10.3389/fimmu.2021.731845 34616400 PMC8488440

[135] Proft T Fraser JD. Bacterial superantigens. Clin Exp Immunol. (2003) 133:299–306. doi: 10.1046/j.1365-2249.2003.02203.x 12930353 PMC1808794

[136] Heinzel S Marchingo JM Horton MB Hodgkin PD. The regulation of lymphocyte activation and proliferation. Curr Opin Immunol. (2018) 51:32–8. doi: 10.1016/j.coi.2018.01.002 29414529

[137] Li KP Shanmuganad S Carroll K Katz JD Jordan MB Hildeman DA. Dying to protect: cell death and the control of T-cell homeostasis. Immunol Rev. (2017) 277:21–43. doi: 10.1111/imr.12538 28462527 PMC5416827

[138] Arakaki R Yamada A Kudo Y Hayashi Y Ishimaru N. Mechanism of activation-induced cell death of T cells and regulation of FasL expression. Crit Rev Immunol. (2014) 34:301–14. doi: 10.1615/critrevimmunol.2014009988 24941158

[139] Martin MD Badovinac VP. Antigen-dependent and -independent contributions to primary memory CD8 T cell activation and protection following infection. Sci Rep. (2015) 5:18022. doi: 10.1038/srep18022 26658291 PMC4675085

[140] Barnaba V. T cell memory in infection, cancer, and autoimmunity. Front Immunol. (2022) 12:811968. doi: 10.3389/fimmu.2021.811968 35069600 PMC8771143

[141] Li Y Li Z Hu F. Double-negative (DN) B cells: an under-recognized effector memory B cell subset in autoimmunity. Clin Exp Immunol. (2021) 205:119–27. doi: 10.1111/cei.13615 PMC8274172 33969476

[142] Klammer H Mladenov E Li F Iliakis G. Bystander effects as manifestation of intercellular communication of DNA damage and of the cellular oxidative status. Cancer Lett. (2015) 356:58–71. doi: 10.1016/j.canlet.2013.12.017 24370566

[143] Mladenov E Li F Zhang L Klammer H Iliakis G. Intercellular communication of DNA damage and oxidative status underpin bystander effects. Int J Radiat Biol. (2018) 94:719–26. doi: 10.1080/09553002.2018.1434323 29377786

[144] Lin D Medeiros DM. The microbiome as a major function of the gastrointestinal tract and its implication in micronutrient metabolism and chronic diseases. Nutr Res. (2023) 112:30–45. doi: 10.1016/j.nutres.2023.02.007 36965327

[145] Ghorbaninejad M Asadzadeh-Aghdaei H Baharvand H Meyfour A. Intestinal organoids: A versatile platform for modeling gastrointestinal diseases and monitoring epigenetic alterations. Life Sci. (2023) 319:121506. doi: 10.1016/j.lfs.2023.121506 36858311

[146] Yoo JY Groer M Dutra SVO Sarkar A McSkimming DI. Gut microbiota and immune system interactions. Microorganisms. (2020) 8:1587. doi: 10.3390/microorganisms8101587 33076307 PMC7602490

[147] Shim JA Ryu JH Jo Y Hong C. The role of gut microbiota in T cell immunity and immune mediated disorders. Int J Biol Sci. (2023) 19:1178–91. doi: 10.7150/ijbs.79430 PMC10008692 36923929

[148] Takiishi T Fenero CIM Câmara NOS. Intestinal barrier and gut microbiota: Shaping our immune responses throughout life. Tissue Barriers. (2017) 5:e1373208. doi: 10.1080/21688370.2017.1373208 28956703 PMC5788425

[149] Miyakawa M Iijima S Kobayashi R Tajima M. Observation on the lymphoid tissue of the germ-free Guinea pig. Pathol Int. (1957) 7:183–210. doi: 10.1111/j.1440-1827.1957.tb00483.x

[150] Alexander TJ Miniats OP Ingram DG Thomson RG Thackeray EL. Gnotobiotic pigs: procurement, microbial flora, serum proteins and lymphatic tissues. Can Vet J. (1969) 10:98–105. 4181892 PMC1697446

[151] Bauer H Horowitz RE Levenson SM Popper H. The response of the lymphatic tissue to the microbial flora. Studies on germfree mice. Am J Pathol. (1963) 42:471–83. PMC1949649 13966929

[152] Mousa WK Chehadeh F Husband S. Microbial dysbiosis in the gut drives systemic autoimmune diseases. Front Immunol. (2022) 13:906258. doi: 10.3389/fimmu.2022.906258 36341463 PMC9632986

[153] Miyauchi E Shimokawa C Steimle A Desai MS Ohno H. The impact of the gut microbiome on extra-intestinal autoimmune diseases. Nat Rev Immunol. (2023) 23:9–23. doi: 10.1038/s41577-022-00727-y 35534624

[154] Rogler G Singh A Kavanaugh A Rubin DT. Extraintestinal manifestations of inflammatory bowel disease: current concepts, treatment, and implications for disease management. Gastroenterology. (2021) 166:1118–32. doi: 10.1053/j.gastro.2021.07.042 PMC8564770 34358489

[155] Greuter T Vavricka SR. Extraintestinal manifestations in inflammatory bowel disease - epidemiology, genetics, and pathogenesis. Expert Rev Gastroenterol Hepatol. (2019) 13:307–17. doi: 10.1080/17474124.2019.1574569 30791773

[156] Taurog JD Richardson JA Croft JT Simmons WA Zhou M Fernández-Sueiro JL. The germfree state prevents development of gut and joint inflammatory disease in HLA-B27 transgenic rats. J Exp Med. (1994) 180:2359–64. doi: 10.1084/jem.180.6.2359 PMC2191772 7964509

[157] Maeda Y Kurakawa T Umemoto E Motooka D Ito Y Gotoh K. Dysbiosis contributes to arthritis development via activation of autoreactive T cells in the intestine. Arthritis Rheumatol. (2016) 68:2646–61. doi: 10.1002/art.39783 27333153

[158] Rehaume LM Mondot S Aguirre de Cárcer D Velasco J Benham H Hasnain SZ. ZAP-70 genotype disrupts the relationship between microbiota and host, leading to spondyloarthritis and ileitis in SKG mice. Arthritis Rheumatol. (2014) 66:2780–92. doi: 10.1002/art.38773 25048686

[159] Berer K Mues M Koutrolos M Rasbi ZA Boziki M Johner C. Commensal microbiota and myelin autoantigen cooperate to trigger autoimmune demyelination. Nature. (2011) 479:538–41. doi: 10.1038/nature10554 22031325

[160] Luce S Guinoiseau S Gadault A Letourneur F Blondeau B Nitschke P. Humanized mouse model to study type 1 diabetes. Diabetes. (2018) 67:1816–29. doi: 10.2337/db18-0202 29967002

[161] Heidt PJ Vossen JM. Experimental and clinical gnotobiotics: influence of the microflora on graft-versus-host disease after allogeneic bone marrow transplantation. J Med. (1992) 23:161–73. 1479298

[162] Vita AA Aljobaily H Lyons DO Pullen NA. Berberine delays onset of collagen-induced arthritis through T cell suppression. Int J Mol Sci. (2021) 22:3522. doi: 10.3390/ijms22073522 33805383 PMC8037694

[163] Shen L. Functional morphology of the gastrointestinal tract. Curr Top Microbiol Immunol. (2009) 337:1–35. doi: 10.1007/978-3-642-01846-6_1 19812978

[164] Guven-Maiorov E Tsai CJ Nussinov R. Structural host-microbiota interaction networks. PloS Comput Biol. (2017) 13:e1005579. doi: 10.1371/journal.pcbi.1005579 29023448 PMC5638203

[165] Gomez A Sharma AK Mallott EK Petrzelkova KJ Jost Robinson CA Yeoman CJ. Plasticity in the human gut microbiome defies evolutionary constraints. mSphere. (2019) 4:e00271–19. doi: 10.1128/mSphere.00271-19 PMC6669335 31366708

[166] Rothschild D Weissbrod O Barkan E Kurilshikov A Korem T Zeevi D. Environment dominates over host genetics in shaping human gut microbiota. Nature. (2018) 555:210–5. doi: 10.1038/nature25973 29489753

[167] Dehghani T Gholizadeh O Daneshvar M Nemati MM Akbarzadeh S Amini P. Association between inflammatory bowel disease and viral infections. Curr Microbiol. (2023) 80:195. doi: 10.1007/s00284-023-03305-0 37106245 PMC10139670

[168] Zhang L Liu F Xue J Lee SA Liu L Riordan SM. Bacterial species associated with human inflammatory bowel disease and their pathogenic mechanisms. Front Microbiol. (2022) 13:801892. doi: 10.3389/fmicb.2022.801892 35283816 PMC8908260

[169] Gomes-Neto JC Pavlovikj N Korth N Naberhaus SA Arruda B Benson AK. Salmonella enterica induces biogeography-specific changes in the gut microbiome of pigs. Front Vet Sci. (2023) 10:1186554. doi: 10.3389/fvets.2023.1186554 37781286 PMC10537282

[170] Feilstrecker Balani G Dos Santos Cortez M Picasky da Silveira Freitas JE Freire de Melo F Zarpelon-Schutz AC Teixeira KN. Immune response modulation in inflammatory bowel diseases by Helicobacter pylori infection. World J Gastroenterol. (2023) 29:4604–15. doi: 10.3748/wjg.v29.i30.4604 PMC10472898 37662864

[171] Chen X Wang S Mao X Xiang X Ye S Chen J. Adverse health effects of emerging contaminants on inflammatory bowel disease. Front Public Health. (2023) 11:1140786. doi: 10.3389/fpubh.2023.1140786 36908414 PMC9999012

[172] Eichele DD Kharbanda KK. Dextran sodium sulfate colitis murine model: An indispensable tool for advancing our understanding of inflammatory bowel diseases pathogenesis. World J Gastroenterol. (2017) 23:6016–29. doi: 10.3748/wjg.v23.i33.6016 PMC5597494 28970718

[173] Yoon MY Yoon SS. Disruption of the gut ecosystem by antibiotics. Yonsei Med J. (2018) 59:4–12. doi: 10.3349/ymj.2018.59.1.4 29214770 PMC5725362

[174] Wang X Tang Q Hou H Zhang W Li M Chen D. Gut microbiota in NSAID enteropathy: new insights from inside. Front Cell Infect Microbiol. (2021) 11:679396. doi: 10.3389/fcimb.2021.679396 34295835 PMC8290187

[175] He Y Zheng J Ye B Dai Y Nie K. Chemotherapy-induced gastrointestinal toxicity: Pathogenesis and current management. Biochem Pharmacol. (2023) 216:115787. doi: 10.1016/j.bcp.2023.115787 37666434

[176] David LA Maurice CF Carmody RN Gootenberg DB Button JE Wolfe BE. Diet rapidly and reproducibly alters the human gut microbiome. Nature. (2014) 505:559–63. doi: 10.1038/nature12820 PMC3957428 24336217

[177] Gill PA Inniss S Kumagai T Rahman FZ Smith AM. The role of diet and gut microbiota in regulating gastrointestinal and inflammatory disease. Front Immunol. (2022) 13:866059. doi: 10.3389/fimmu.2022.866059 35450067 PMC9016115

[178] Yang W Cong Y. Gut microbiota-derived metabolites in the regulation of host immune responses and immune-related inflammatory diseases. Cell Mol Immunol. (2021) 18:866–77. doi: 10.1038/s41423-021-00661-4 PMC8115644 33707689

[179] Lu Y Li Z Peng X. Regulatory effects of oral microbe on intestinal microbiota and the illness. Front Cell Infect Microbiol. (2023) 13:1093967. doi: 10.3389/fcimb.2023.1093967 36816583 PMC9928999

[180] du Teil Espina M Gabarrini G Harmsen HJM Westra J van Winkelhoff AJ van Dijl JM. Talk to your gut: the oral-gut microbiome axis and its immunomodulatory role in the etiology of rheumatoid arthritis. FEMS Microbiol Rev. (2019) 43:1–18. doi: 10.1093/femsre/fuy035 30219863

[181] Yoon H Park YS Shin CM Kim N Lee DH. Gut microbiome in probable intestinal tuberculosis and changes following anti-tuberculosis treatment. Yonsei Med J. (2022) 63:34–41. doi: 10.3349/ymj.2022.63.1.34 34913282 PMC8688367

[182] Haneishi Y Furuya Y Hasegawa M Picarelli A Rossi M Miyamoto J. Inflammatory bowel diseases and gut microbiota. Int J Mol Sci. (2023) 24:3817. doi: 10.3390/ijms24043817 36835245 PMC9958622

[183] Saez A Herrero-Fernandez B Gomez-Bris R Sánchez-Martinez H Gonzalez-Granado JM. Pathophysiology of inflammatory bowel disease: innate immune system. Int J Mol Sci. (2023) 24:1526. doi: 10.3390/ijms24021526 36675038 PMC9863490

[184] Jiao Y Wu L Huntington ND Zhang X. Crosstalk between gut microbiota and innate immunity and its implication in autoimmune diseases. Front Immunol. (2020) 11:282. doi: 10.3389/fimmu.2020.00282 32153586 PMC7047319

[185] Littman DR Pamer EG. Role of the commensal microbiota in normal and pathogenic host immune responses. Cell Host Microbe. (2011) 10:311–23. doi: 10.1016/j.chom.2011.10.004 PMC3202012 22018232

[186] Eloe-Fadrosh EA Rasko DA. The human microbiome: from symbiosis to pathogenesis. Annu Rev Med. (2013) 64:145–63. doi: 10.1146/annurev-med-010312-133513 PMC3731629 23327521

[187] Talipova D Smagulova A Poddighe D. Toll-like receptors and celiac disease. Int J Mol Sci. (2022) 24:265. doi: 10.3390/ijms24010265 36613709 PMC9820541

[188] Jensen SK Pærregaard SI Brandum EP Jørgensen AS Hjortø GM Jensen BAH. Rewiring host-microbe interactions and barrier function during gastrointestinal inflammation. Gastroenterol Rep (Oxf). (2022) 10:goac008. doi: 10.1093/gastro/goac008 35291443 PMC8915887

[189] Brown EM Kenny DJ Xavier RJ. Gut microbiota regulation of T cells during inflammation and autoimmunity. Annu Rev Immunol. (2019) 37:599–624. doi: 10.1146/annurev-immunol-042718-041841 31026411

[190] Weis AM Soto R Round JL. Commensal regulation of T cell survival through Erdr1. Gut Microbes. (2018) 9:458–64. doi: 10.1080/19490976.2018.1441662 PMC6219650 29543554

[191] Zouali M. B lymphocytes, the gastrointestinal tract and autoimmunity. Autoimmun Rev. (2021) 20:102777. doi: 10.1016/j.autrev.2021.102777 33609796

[192] Khatri V Chauhan N Kalyanasundaram R. Parasite cystatin: immunomodulatory molecule with therapeutic activity against immune mediated disorders. Pathogens. (2020) 9:431. doi: 10.3390/pathogens9060431 32486220 PMC7350340

[193] Rasouli-Saravani A Jahankhani K Moradi S Gorgani M Shafaghat Z Mirsanei Z. Role of microbiota short-chain fatty acids in the pathogenesis of autoimmune diseases. BioMed Pharmacother. (2023) 162:114620. doi: 10.1016/j.biopha.2023.114620 37004324

[194] Su X Gao Y Yang R. Gut microbiota derived bile acid metabolites maintain the homeostasis of gut and systemic immunity. Front Immunol. (2023) 14:1127743. doi: 10.3389/fimmu.2023.1127743 37256134 PMC10225537

[195] Brown J Robusto B Morel L. Intestinal dysbiosis and tryptophan metabolism in autoimmunity. Front Immunol. (2020) 11:1741. doi: 10.3389/fimmu.2020.01741 32849620 PMC7417361

[196] Cheng Z Wang Y Li B. Dietary polyphenols alleviate autoimmune liver disease by mediating the intestinal microenvironment: challenges and hopes. J Agric Food Chem. (2022) 70:10708–37. doi: 10.1021/acs.jafc.2c02654 36005815

[197] Ma X Fan PX Li LS Qiao SY Zhang GL Li DF. Butyrate promotes the recovering of intestinal wound healing through its positive effect on the tight junctions. J Anim Sci. (2012) 90 Suppl 4:266–8. doi: 10.2527/jas.50965 23365351

[198] Nakano T Uchiyama K Ushiroda C Kashiwagi S Toyokawa Y Mizushima K. Promotion of wound healing by acetate in murine colonic epithelial cell via c-Jun N-terminal kinase activation. J Gastroenterol Hepatol. (2020) 35:1171–79. doi: 10.1111/jgh.14987 31961456

[199] Feng Y Wang Y Wang P Huang Y Wang F. Short-chain fatty acids manifest stimulative and protective effects on intestinal barrier function through the inhibition of NLRP3 inflammasome and autophagy. Cell Physiol Biochem. (2018) 49:190–205. doi: 10.1159/000492853 30138914

[200] Wang RX Lee JS Campbell EL Colgan SP. Microbiota-derived butyrate dynamically regulates intestinal homeostasis through regulation of actin- associated protein synaptopodin. Proc Natl Acad Sci U.S.A. (2020) 117:11648–57. doi: 10.1073/pnas.1917597117 PMC7260972 32398370

[201] Vogelzang A Guerrini MM Minato N Fagarasan S. Microbiota - an amplifier of autoimmunity. Curr Opin Immunol. (2018) 55:15–21. doi: 10.1016/j.coi.2018.09.003 30248521

[202] Li R Meng X Chen B Zhao L Zhang X. Gut microbiota in lupus: a butterfly effect? Curr Rheumatol Rep. (2021) 23:27. doi: 10.1007/s11926-021-00986-z 33864162

[203] Zhang H Liu M Zhong W Zheng Y Li Y Guo L. Leaky gut driven by dysbiosis augments activation and accumulation of liver macrophages via RIP3 signaling pathway in autoimmune hepatitis. Front Immunol. (2021) 12:624360. doi: 10.3389/fimmu.2021.624360 33841405 PMC8027109

[204] Costa MC Santos JR Ribeiro MJ Freitas GJ Bastos RW Ferreira GF. The absence of microbiota delays the inflammatory response to Cryptococcus gattii. Int J Med Microbiol. (2016) 306:187–95. doi: 10.1016/j.ijmm.2016.03.010 27083265

[205] Ost KS Round JL. A few good commensals: gut microbes use IFN-γ to fight salmonella. Immunity. (2017) 46:977–79. doi: 10.1016/j.immuni.2017.06.010 28636967

[206] SahBandar IN Chew GM Corley MJ Pang APS Tsai N Hanks N. Changes in gastrointestinal microbial communities influence HIV-specific CD8+ T-cell responsiveness to immune checkpoint blockade. AIDS. (2020) 34:1451–60. doi: 10.1097/QAD.0000000000002557 PMC7371239 32675558

[207] Hong SH. Influence of microbiota on vaccine effectiveness: “Is the microbiota the key to vaccine-induced responses?” J Microbiol. (2023) 61:483–94. doi: 10.1007/s12275-023-00044-6 PMC10098251 37052795

[208] Stražar M Mourits VP Koeken VACM de Bree LCJ Moorlag SJCFM Joosten LAB. The influence of the gut microbiome on BCG-induced trained immunity. Genome Biol. (2021) 22:275. doi: 10.1186/s13059-021-02482-0 34551799 PMC8456614

[209] Dow CT. Proposing BCG Vaccination for Mycobacterium avium. paratuberculosis (MAP) Associated Autoimmune Diseases. Microorganisms. (2020) 8:212. doi: 10.3390/microorganisms8020212 32033287 PMC7074941

[210] Goudouris E Aranda CS Solé D. Implications of the non-specific effect induced by Bacillus Calmette-Guerin (BCG) vaccine on vaccine recommendations. J Pediatr (Rio J). (2023) 99 Suppl 1:S22–7. doi: 10.1016/j.jped.2022.09.002 PMC10066422 36309066

[211] Noh ASM Chuan TD Khir NAM Zin AAM Ghazali AK Long I. Effects of different doses of complete Freund’s adjuvant on nociceptive behaviour and inflammatory parameters in polyarthritic rat model mimicking rheumatoid arthritis. PloS One. (2021) 16:e0260423. doi: 10.1371/journal.pone.0260423 34879087 PMC8654228

[212] Billiau A Matthys P. Modes of action of Freund’s adjuvants in experimental models of autoimmune diseases. J Leukoc Biol. (2001) 70:849–60. 11739546

[213] Kuroda Y Nacionales DC Akaogi J Reeves WH Satoh M. Autoimmunity induced by adjuvant hydrocarbon oil components of vaccine. BioMed Pharmacother. (2004) 58:325–37. doi: 10.1016/j.biopha.2004.04.009 15194169

[214] Seida I Seida R Elsalti A Mahroum N. Vaccines and autoimmunity-from side effects to ASIA syndrome. Medicina (Kaunas). (2023) 59:364. doi: 10.3390/medicina59020364 36837564 PMC9966463

[215] Segal Y Dahan S Sharif K Bragazzi NL Watad A Amital H. The value of Autoimmune Syndrome Induced by Adjuvant (ASIA) - Shedding light on orphan diseases in autoimmunity. Autoimmun Rev. (2018) 17:440–8. doi: 10.1016/j.autrev.2017.11.037 29526630

[216] Jara LJ García-Collinot G Medina G Cruz-Dominguez MDP Vera-Lastra O Carranza-Muleiro RA. Severe manifestations of autoimmune syndrome induced by adjuvants (Shoenfeld’s syndrome). Immunol Res. (2017) 65:8–16. doi: 10.1007/s12026-016-8811-0 27412294

[217] Jørgensen TN Thurman J Izui S Falta MT Metzger TE Flannery SA. Genetic susceptibility to polyI:C-induced IFNalpha/beta-dependent accelerated disease in lupus-prone mice. Genes Immun. (2006) 7:555–67. doi: 10.1038/sj.gene.6364329 16900204

[218] Shi YN Liu FH Yu XJ Liu ZB Li QX Yuan JH. Polyinosine- polycytidylic acid promotes excessive iodine intake induced thyroiditis in non-obese diabetic mice via Toll-like receptor 3 mediated inflammation. Chin (Engl). (2013) 126:703–10. doi: 10.3760/cma.j.issn.0366-6999.20122723 23422193

[219] Udompornpitak K Bhunyakarnjanarat T Charoensappakit A Dang CP Saisorn W Leelahavanichkul A. Lipopolysaccharide-enhanced responses against aryl hydrocarbon receptor in fcgRIIb-deficient macrophages, a profound impact of an environmental toxin on a lupus-like mouse model. Int J Mol Sci. (2021) 22:4199. doi: 10.3390/ijms22084199 33919603 PMC8073880

[220] Kasperkiewicz K Świerzko AS Przybyła M Szemraj J Barski J Skurnik M. The role of yersinia enterocolitica O:3 lipopolysaccharide in collagen-induced arthritis. J Immunol Res. (2020) 2020:7439506. doi: 10.1155/2020/7439506 33274243 PMC7676966

[221] Nogai A Siffrin V Bonhagen K Pfueller CF Hohnstein T Volkmer-Engert R. Lipopolysaccharide injection induces relapses of experimental autoimmune encephalomyelitis in nontransgenic mice via bystander activation of autoreactive CD4+ cells. J Immunol. (2005) 175:959–66. doi: 10.4049/jimmunol.175.2.959 16002695

[222] Yoshitomi H Sakaguchi N Kobayashi K Brown GD Tagami T Sakihama T. A role for fungal {beta}-glucans and their receptor Dectin-1 in the induction of autoimmune arthritis in genetically susceptible mice. J Exp Med. (2005) 201:949–60. doi: 10.1084/jem.20041758 PMC2213107 15781585

[223] Kohashi O Kuwata J Umehara K Uemura F Takahashi T Ozawa A. Susceptibility to adjuvant-induced arthritis among germfree, specific-pathogen-free, and conventional rats. Infect Immun. (1979) 26:791–4. doi: 10.1128/iai.26.3.791-794.1979 PMC414687 160888

[224] Tanaka A Saito R Sugiyama K Morisaki I Kotani S. Adjuvant activity of synthetic N-acetylmuramyl peptides in rats. Infect Immun. (1977) 15:332–4. doi: 10.1128/iai.15.1.332-334.1977 PMC421367 832904

[225] Fang J Fang D Silver PB Wen F Li B Ren X. The role of TLR2, TRL3, TRL4, and TRL9 signaling in the pathogenesis of autoimmune disease in a retinal autoimmunity model. Invest Ophthalmol Vis Sci. (2010) 51:3092–9. doi: 10.1167/iovs.09-4754 PMC2891468 20107166

[226] Abdollahi-Roodsaz S Joosten LA Koenders MI Devesa I Roelofs MF Radstake TR. Stimulation of TLR2 and TLR4 differentially skews the balance of T cells in a mouse model of arthritis. J Clin Invest. (2008) 118:205–16. doi: 10.1172/JCI32639 PMC2104479 18060042

[227] Haanen J Ernstoff MS Wang Y Menzies AM Puzanov I Grivas P. Autoimmune diseases and immune-checkpoint inhibitors for cancer therapy: review of the literature and personalized risk-based prevention strategy. Ann Oncol. (2020) 31:724–44. doi: 10.1016/j.annonc.2020.03.285 32194150

[228] Ceccarelli F Natalucci F Picciariello L Olivieri G Cirillo A Gelibter A. Rheumatic diseases development in patients treated by anti-PD1 immune checkpoint inhibitors: A single-centre descriptive study. Life (Basel). (2023) 13:877. doi: 10.3390/life13040877 37109406 PMC10141051

[229] Harada T Ohno N. Contribution of dectin-1 and granulocyte macrophage-colony stimulating factor (GM-CSF) to immunomodulating actions of beta-glucan. Int Immunopharmacol. (2008) 8:556–66. doi: 10.1016/j.intimp.2007.12.011 18328447

[230] Hida TH Kawaminami H Ishibashi K Miura NN Adachi Y Yadomae T. Effect of GM-CSF on cytokine induction by soluble beta-glucan SCG in *vitro* in beta-glucan-treated mice. Microbiol Immunol. (2009) 53:391–402. doi: 10.1111/j.1348-0421.2009.00139.x 19563398

[231] Nissen C Stern M. Acquired immune mediated aplastic anemia: is it antineoplastic? Autoimmun Rev. (2009) 9:11–6. doi: 10.1016/j.autrev.2009.02.032 19245859

[232] Purev E Dumitriu B Hourigan CS Young NS Townsley DM. Translocation (8;21) acute myeloid leukemia presenting as severe aplastic anemia. Leuk Res Rep. (2014) 3:46–8. doi: 10.1016/j.lrr.2014.04.002 PMC4081983 25003026

[233] Pęczek P Gajda M Rutkowski K Fudalej M Deptała A Badowska-Kozakiewicz AM. Cancer-associated inflammation: pathophysiology and clinical significance. J Cancer Res Clin Oncol. (2023) 149:2657–72. doi: 10.1007/s00432-022-04399-y PMC9579684 36260158

[234] Sahu A Kose K Kraehenbuehl L Byers C Holland A Tembo T. *In vivo* tumor immune microenvironment phenotypes correlate with inflammation and vasculature to predict immunotherapy response. Nat Commun. (2022) 13:5312. doi: 10.1038/s41467-022-32738-7 36085288 PMC9463451

[235] Wheeler OPG Unterholzner L. DNA sensing in cancer: Pro-tumour and anti- tumour functions of cGAS-STING signalling. Essays Biochem. (2023) 67:905–18. doi: 10.1042/EBC20220241 PMC10539950 37534795

[236] Kouroukli O Symeonidis A Foukas P Maragkou MK Kourea EP. Bone marrow immune microenvironment in myelodysplastic syndromes. Cancers (Basel). (2022) 14:5656. doi: 10.3390/cancers14225656 36428749 PMC9688609

[237] Vallelonga V Gandolfi F Ficara F Della Porta MG Ghisletti S. Emerging insights into molecular mechanisms of inflammation in myelodysplastic syndromes. Biomedicines. (2023) 11:2613. doi: 10.3390/biomedicines11102613 37892987 PMC10603842

[238] Hashimoto H Güngör D Krickeberg N Schmitt J Doll L Schmidt M. TH1 cytokines induce senescence in AML. Leuk Res. (2022) 117:106842. doi: 10.1016/j.leukres.2022.106842 35490594

[239] Mucci A Antonarelli G Caserta C Vittoria FM Desantis G Pagani R. Myeloid cell-based delivery of IFN-γ reprograms the leukemia microenvironment and induces anti-tumoral immune responses. EMBO Mol Med. (2021) 13:e13598. doi: 10.15252/emmm.202013598 34459560 PMC8495462

[240] Votavová H Lenertová Z Votava T Beličková M. Hypoplastic form of myelodysplastic neoplasm. Klin Onkol. (2023) 36:206–14. doi: 10.48095/ccko2023206 37353349

[241] Rosenberg PS Alter BP Bolyard AA Bonilla MA Boxer LA Cham B. The incidence of leukemia and mortality from sepsis in patients with severe congenital neutropenia receiving long-term G-CSF therapy. Blood. (2006) 107:4628–35. doi: 10.1182/blood-2005-11-4370 PMC1895804 16497969

[242] Newburger PE Dale DC. Evaluation and management of patients with isolated neutropenia. Semin Hematol. (2013) 50:198–206. doi: 10.1053/j.seminhematol.2013.06.010 23953336 PMC3748385

[243] Sung L Aplenc R Alonzo TA Gerbing RB Wang YC Meshinchi S. Association between prolonged neutropenia and reduced relapse risk in pediatric AML: A report from the children’s oncology group. Int J Cancer. (2016) 139:1930–5. doi: 10.1002/ijc.30236 PMC4990479 27312107

[244] Aoki T Takahashi H Tanaka S Shiba N Hasegawa D Iwamoto S. Predisposition to prolonged neutropenia after chemotherapy for paediatric acute myeloid leukaemia is associated with better prognosis in the Japanese Paediatric Leukaemia/Lymphoma Study Group AML-05 study. Br J Haematol. (2021) 193:176–80. doi: 10.1111/bjh.16656 32337716

[245] Imataki O Ishida T Kida JI Uemura M Fujita H Kadowaki N. Repeated spontaneous remission of acute myeloid leukemia in response to various infections: a case report. BMC Infect Dis. (2023) 23:215. doi: 10.1186/s12879-023-08108-z 37024850 PMC10080930

[246] Kandeel EZ Refaat L Abdel-Fatah R Samra M Bayoumi A Abdellateif MS. Could COVID-19 induce remission of acute leukemia? Hematology. (2021) 26:870–3. doi: 10.1080/16078454.2021.1992117 34719343

[247] Takamatsu Y Miyamoto T Iwasaki H Makino S Tamura K. Remission induction by granulocyte colony-stimulating factor in hypoplastic acute myelogenous leukemia complicated by infection. A Case Rep Rev literature. Acta Haematol. (1998) 99:224–30. doi: 10.1159/000040844 9644302

[248] Trof RJ Beishuizen A Wondergem MJ Strack van Schijndel RJ. Spontaneous remission of acute myeloid leukaemia after recovery from sepsis. Neth J Med. (2007) 65:259–62. 17656812

[249] Maywald O Buchheidt D Bergmann J Schoch C Ludwig WD Reiter A. Spontaneous remission in adult acute myeloid leukemia in association with systemic bacterial infection-case report and review of the literature. Ann Hematol. (2004) 83:189–94. doi: 10.1007/s00277-003-0741-y 15064869

[250] You L Liu Y Chen N Zhu L Xu G Lv Z. (H1N1) virus induced long-term remission in a refractory acute myeloid leukaemia. Br J Haematol. (2023) 202:745–8. doi: 10.1111/bjh.18876 37230766

[251] Jimemez C Ribera JM Abad E Pintos G Milla F Junca J. Increased serum tumour necrosis factor during transient remission in acute leukaemia. Lancet. (1993) 341:1600. doi: 10.1016/0140-6736(93)90739-4 8099677

[252] Musto P D’Arena G Melillo L Cascavilla N La Sala A Ladogana S. Spontaneous remission in acute myeloid leukaemia: a role for endogenous production of tumour necrosis factor and interleukin-2? Br J Haematol. (1994) 87:879–80. doi: 10.1111/j.1365-2141.1994.tb06761.x 7986737

[253] Müller-Schmah C Solari L Weis R Pfeifer D Scheibenbogen C Trepel M. Immune response as a possible mechanism of long-lasting disease control in spontaneous remission of MLL/AF9-positive acute myeloid leukemia. Ann Hematol. (2012) 91:27–32. doi: 10.1007/s00277-011-1332-y 21959947

[254] Grunwald VV Hentrich M Schiel X Dufour A Schneider S Neusser M. Patients with spontaneous remission of high-risk MDS and AML show persistent preleukemic clonal hematopoiesis. Blood Adv. (2019) 3:2696–9. doi: 10.1182/bloodadvances.2019000265 PMC6759731 31515231

[255] Coulie PG Van den Eynde BJ van der Bruggen P Boon T. Tumour antigens recognized by T lymphocytes: at the core of cancer immunotherapy. Nat Rev Cancer. (2014) 14:135–46. doi: 10.1038/nrc3670 24457417

[256] O’Reilly E Zeinabad HA Szegezdi E. Hematopoietic versus leukemic stem cell quiescence: Challenges and therapeutic opportunities. Blood Rev. (2021) 50:100850. doi: 10.1016/j.blre.2021.100850 34049731

[257] Sahin U Türeci Ö. Personalized vaccines for cancer immunotherapy. Science. (2018) 359:1355–60. doi: 10.1126/science.aar7112 29567706

[258] Greiner J Ringhoffer M Simikopinko O Szmaragowska A Huebsch S Maurer U. Simultaneous expression of different immunogenic antigens in acute myeloid leukemia. Exp Hematol. (2000) 28:1413–22. doi: 10.1016/s0301-472x(00)00550-6 11146163

[259] Healy FM Dahal LN Jones JRE Floisand Y Woolley JF. Recent progress in interferon therapy for myeloid Malignancies. Front Oncol. (2021) 11:769628. doi: 10.3389/fonc.2021.769628 34778087 PMC8586418

[260] Kiladjian JJ Giraudier S Cassinat B. Interferon-alpha for the therapy of myeloproliferative neoplasms: targeting the Malignant clone. Leukemia. (2016) 30:776–81. doi: 10.1038/leu.2015.326 26601783

[261] Fan S Pan TZ Dou LP Zhao YM Zhang XH Xu LP. Preemptive interferon-α therapy could prevent relapse of acute myeloid leukemia following allogeneic hematopoietic stem cell transplantation: A real-world analysis. Front Immunol. (2023) 14:1091014. doi: 10.3389/fimmu.2023.1091014 36817493 PMC9932895

[262] Li X Yang Y Yuan J Hong P Freie B Orazi A. Continuous in *vivo* infusion of interferon-gamma (IFN-gamma) preferentially reduces myeloid progenitor numbers and enhances engraftment of syngeneic wild- type cells in Fancc-/- mice. Blood. (2004) 104:1204–9. doi: 10.1182/blood-2004-03-1094 15113761

[263] Glavan TM Pavelic J. The exploitation of Toll-like receptor 3 signaling in cancer therapy. Curr Pharm Des. (2014) 20:6555–64. doi: 10.2174/1381612820666140826153347 25341932

[264] Bianchi F Pretto S Tagliabue E Balsari A Sfondrini L. Exploiting poly(I:C) to induce cancer cell apoptosis. Cancer Biol Ther. (2017) 18:747–56. doi: 10.1080/15384047.2017.1373220 PMC5678690 28881163

[265] Lion E Anguille S Berneman ZN Smits EL Van Tendeloo VF. Poly(I:C) enhances the susceptibility of leukemic cells to NK cell cytotoxicity and phagocytosis by DC. PloS One. (2011) 6:e20952. doi: 10.1371/journal.pone.0020952 21698118 PMC3117863

[266] Cardillo F Bonfim M da Silva Vasconcelos Sousa P Mengel J Ribeiro Castello-Branco LR Pinho RT. Bacillus calmette-guérin immunotherapy for cancer. Vaccines (Basel). (2021) 9:439. doi: 10.3390/vaccines9050439 34062708 PMC8147207

[267] Reizenstein P. Adjuvant immunotherapy with BCG of acute myeloid leukemia: a 15-year follow-up. Br J Haematol. (1990) 75:288–9. doi: 10.1111/j.1365-2141.1990.tb02667.x 2196933

[268] Morra ME Kien ND Elmaraezy A Abdelaziz OAM Elsayed AL Halhouli O. Early vaccination protects against childhood leukemia: A systematic review and meta-analysis. Sci Rep. (2017) 7:15986. doi: 10.1038/s41598-017-16067-0 29167460 PMC5700199

[269] Hjelholt AJ Bergh C Bhatt DL Fröbert O Kjolby MF. Pleiotropic effects of influenza vaccination. Vaccines (Basel). (2023) 11:1419. doi: 10.3390/vaccines11091419 37766096 PMC10536538

[270] Aznar MA Molina C Teijeira A Rodriguez I Azpilikueta A Garasa S. Repurposing the yellow fever vaccine for intratumoral immunotherapy. EMBO Mol Med. (2020) 12:e10375. doi: 10.15252/emmm.201910375 31746149 PMC6949490

[271] Deldadeh N Haghighat S Omidi Z Sarrami-Foroushani R Ansari AM Sanati H. Anti-cancer effect of COVID-19 vaccines in mice models. Life Sci. (2023) 325:121569. doi: 10.1016/j.lfs.2023.121569 36907328 PMC10007716

[272] Fang L Liu K Liu C Wang X Ma W Xu W. Tumor accomplice: T cell exhaustion induced by chronic inflammation. Front Immunol. (2022) 13:979116. doi: 10.3389/fimmu.2022.979116 36119037 PMC9479340

[273] Khaldoyanidi S Nagorsen D Stein A Ossenkoppele G Subklewe M. Immune biology of acute myeloid leukemia: implications for immunotherapy. J Clin Oncol. (2021) 39:419–32. doi: 10.1200/JCO.20.00475 PMC8078464 33434043

[274] Abaza Y Zeidan AM. Immune checkpoint inhibition in acute myeloid leukemia and myelodysplastic syndromes. Cells. (2022) 11:2249. doi: 10.3390/cells11142249 35883692 PMC9318025

[275] Greiner J Goetz M Schuler PJ Bulach C Hofmann S Schrezenmeier H. Enhanced stimulation of antigen-specific immune responses against nucleophosmin 1 mutated acute myeloid leukaemia by an anti- programmed death 1 antibody. Br J Haematol. (2022) 198:866–74. doi: 10.1111/bjh.18326 35799423

[276] Ayers M Lunceford J Nebozhyn M Murphy E Loboda A Kaufman DR. IFN-γ-related mRNA profile predicts clinical response to PD-1 blockade. J Clin Invest. (2017) 127:2930–40. doi: 10.1172/JCI91190 PMC5531419 28650338

[277] Lee SE Wang F Grefe M Trujillo-Ocampo A Ruiz-Vasquez W Takahashi K. Immunologic predictors for clinical responses during immune checkpoint blockade in patients with myelodysplastic syndromes. Clin Cancer Res. (2023) 29:1938–51. doi: 10.1158/1078-0432.CCR-22-2601 PMC10192218 36988276

[278] Damei I Trickovic T Mami-Chouaib F Corgnac S. Tumor-resident memory T cells as a biomarker of the response to cancer immunotherapy. Front Immunol. (2023) 14:1205984. doi: 10.3389/fimmu.2023.1205984 37545498 PMC10399960

[279] Ott PA Bang YJ Piha-Paul SA Razak ARA Bennouna J Soria JC. T-cell-inflamed gene-expression profile, programmed death ligand 1 expression, and tumor mutational burden predict efficacy in patients treated with pembrolizumab across 20 cancers: KEYNOTE-028. J Clin Oncol. (2019) 37:318–27. doi: 10.1200/JCO.2018.78.2276 30557521

[280] Xu Y Chen Y Niu Z Xing J Yang Z Yin X. A novel pyroptotic and inflammatory gene signature predicts the prognosis of cutaneous melanoma and the effect of anticancer therapies. Front Med (Lausanne). (2022) 9:841568. doi: 10.3389/fmed.2022.841568 35492358 PMC9053829

[281] He Y Huang J Li Q Xia W Zhang C Liu Z. Gut microbiota and tumor immune escape: A new perspective for improving tumor immunotherapy. Cancers (Basel). (2022) 14:5317. doi: 10.3390/cancers14215317 36358736 PMC9656981

[282] DiPalma MP Blattman JN. The impact of microbiome dysbiosis on T cell function within the tumor microenvironment (TME). Front Cell Dev Biol. (2023) 11:1141215. doi: 10.3389/fcell.2023.1141215 37009485 PMC10063789

[283] Luu M Schütz B Lauth M Visekruna A. The impact of gut microbiota-derived metabolites on the tumor immune microenvironment. Cancers (Basel). (2023) 15:1588. doi: 10.3390/cancers15051588 36900377 PMC10001145

[284] Lu Y Yuan X Wang M He Z Li H Wang J. Gut microbiota influence immunotherapy responses: mechanisms and therapeutic strategies. J Hematol Oncol. (2022) 15:47. doi: 10.1186/s13045-022-01273-9 35488243 PMC9052532

[285] Jin Y Dong H Xia L Yang Y Zhu Y Shen Y. The diversity of gut microbiome is associated with favorable responses to anti-programmed death 1 immunotherapy in chinese patients with NSCLC. J Thorac Oncol. (2019) 14:1378–89. doi: 10.1016/j.jtho.2019.04.007 31026576

[286] Li Y Wang S Lin M Hou C Li C Li G. Analysis of interactions of immune checkpoint inhibitors with antibiotics in cancer therapy. Front Med. (2022) 16:307–21. doi: 10.1007/s11684-022-0927-0 35648368

[287] Ferreira A Neves MT Baleiras A Malheiro M Martins A. Fecal microbiota transplant in immunotherapy-resistant melanoma: what can we expect in the near future? Cureus. (2022) 14:e32586. doi: 10.7759/cureus.32586 36654598 PMC9840783

[288] Li Y Elmén L Segota I Xian Y Tinoco R Feng Y. Prebiotic-induced anti-tumor immunity attenuates tumor growth. Cell Rep. (2020) 30:1753–66.e6. doi: 10.1016/j.celrep.2020.01.035 32049008 PMC7053418

[289] Simpson RC Shanahan ER Batten M Reijers ILM Read M Silva IP. Diet-driven microbial ecology underpins associations between cancer immunotherapy outcomes and the gut microbiome. Nat Med. (2022) 28:2344–52. doi: 10.1038/s41591-022-01965-2 36138151

[290] He Y Fu L Li Y Wang W Gong M Zhang J. Gut microbial metabolites facilitate anticancer therapy efficacy by modulating cytotoxic CD8+ T cell immunity. Cell Metab. (2021) 33:988–1000.e7. doi: 10.1016/j.cmet.2021.03.002 33761313

[291] Arrigucci R Lakehal K Vir P Handler D Davidow AL Herrera R. Active tuberculosis is characterized by highly differentiated effector memory th1 cells. Front Immunol. (2018) 9:2127. doi: 10.3389/fimmu.2018.02127 30283456 PMC6156157

[292] Gigley JP Fox BA Bzik DJ. Cell-mediated immunity to Toxoplasma gondii develops primarily by local Th1 host immune responses in the absence of parasite replication. J Immunol. (2009) 182:1069–78. doi: 10.4049/jimmunol.182.2.1069 PMC2615398 19124750

[293] Kurtz JR Goggins JA McLachlan JB. Salmonella infection: Interplay between the bacteria and host immune system. Immunol Lett. (2017) 190:42–50. doi: 10.1016/j.imlet.2017.07.006 28720334 PMC5918639

[294] Singh MK Jamal F Dubey AK Shivam P Kumari S Pushpanjali. Visceral leishmaniasis: A novel nuclear envelope protein ‘nucleoporins-93 (NUP-93)’ from Leishmania donovani prompts macrophage signaling for T-cell activation towards host protective immune response. Cytokine. (2019) 113:200–15. doi: 10.1016/j.cyto.2018.07.005 30001865

[295] Yuan J Xu X Wang Z Tong P Meng X Wu Y. A higher dose of staphylococcus aureus enterotoxin B led to more th1 and lower th2/th1 ratio in th cells. Toxins (Basel). (2023) 15:363. doi: 10.3390/toxins15060363 37368664 PMC10302221

[296] Zhang C Wang J Sun Z Cao Y Mu Z Ji X. Commensal microbiota contributes to predicting the response to immune checkpoint inhibitors in non-small-cell lung cancer patients. Cancer Sci. (2021) 112:3005–17. doi: 10.1111/cas.14979 PMC8353904 34028936

[297] Tanoue T Morita S Plichta DR Skelly AN Suda W Sugiura Y. A defined commensal consortium elicits CD8 T cells and anti-cancer immunity. Nature. (2019) 565:600–5. doi: 10.1038/s41586-019-0878-z 30675064

[298] Chen J Liao W Peng H. Toxoplasma gondii infection possibly reverses host immunosuppression to restrain tumor growth. Front Cell Infect Microbiol. (2022) 12:959300. doi: 10.3389/fcimb.2022.959300 36118042 PMC9470863

[299] Vigneron C Mirouse A Merdji H Rousseau C Cousin C Alby-Laurent F. Sepsis inhibits tumor growth in mice with cancer through Toll-like receptor 4-associated enhanced Natural Killer cell activity. Oncoimmunology. (2019) 8:e1641391. doi: 10.1080/2162402X.2019.1641391 31646090 PMC6791422

[300] Ikeda H Old LJ Schreiber RD. The roles of IFN gamma in protection against tumor development and cancer immunoediting. Cytokine Growth Factor Rev. (2002) 13:95–109. doi: 10.1016/s1359-6101(01)00038-7 11900986

[301] Si W Liang H Bugno J Xu Q Ding X Yang K. Lactobacillus rhamnosus GG induces cGAS/STING- dependent type I interferon and improves response to immune checkpoint blockade. Gut. (2022) 71:521–33. doi: 10.1136/gutjnl-2020-323426 PMC8710942 33685966

[302] Sivan A Corrales L Hubert N Williams JB Aquino-Michaels K Earley ZM. Commensal Bifidobacterium promotes antitumor immunity and facilitates anti-PD-L1 efficacy. Science. (2015) 350:1084–9. doi: 10.1126/science.aac4255 PMC4873287 26541606

[303] Tomita Y Goto Y Sakata S Imamura K Minemura A Oka K. Clostridium butyricum therapy restores the decreased efficacy of immune checkpoint blockade in lung cancer patients receiving proton pump inhibitors. Oncoimmunology. (2022) 11:2081010. doi: 10.1080/2162402X.2022.2081010 35655708 PMC9154751

[304] Kroll MH Rojas-Hernandez C Yee C. Hematologic complications of immune checkpoint inhibitors. Blood. (2022) 139:3594–604. doi: 10.1182/blood.2020009016 PMC9227102 34610113

[305] Filetti M Giusti R Di Napoli A Iacono D Marchetti P. Unexpected serious aplastic anemia from PD-1 inhibitors: beyond what we know. Tumori. (2019) 105:NP48–51. doi: 10.1177/0300891619856197 31221037

[306] O’Connell CL Baer MR Ørskov AD Saini SK Duong VH Kropf P. Safety, outcomes, and T-cell characteristics in patients with relapsed or refractory MDS or CMML treated with atezolizumab in combination with guadecitabine. Clin Cancer Res. (2022) 28:5306–16. doi: 10.1158/1078-0432.CCR-22-1810 PMC9772102 36222848

[307] Chiba Y Mizoguchi I Hasegawa H Ohashi M Orii N Nagai T. Regulation of myelopoiesis by proinflammatory cytokines in infectious diseases. Cell Mol Life Sci. (2018) 75:1363–76. doi: 10.1007/s00018-017-2724-5 PMC11105622 29218601

[308] Boiko JR Borghesi L. Hematopoiesis sculpted by pathogens: Toll-like receptors and inflammatory mediators directly activate stem cells. Cytokine. (2012) 57:1–8. doi: 10.1016/j.cyto.2011.10.005 22079335 PMC3361504

[309] Nagai Y Garrett KP Ohta S Bahrun U Kouro T Akira S. Toll-like receptors on hematopoietic progenitor cells stimulate innate immune system replenishment. Immunity. (2006) 24:801–12. doi: 10.1016/j.immuni.2006.04.008 PMC1626529 16782035

[310] Essers MA Offner S Blanco-Bose WE Waibler Z Kalinke U Duchosal MA. IFNalpha activates dormant haematopoietic stem cells. vivo. Nat. (2009) 458:904–8. doi: 10.1038/nature07815 19212321

[311] Esplin BL Shimazu T Welner RS Garrett KP Nie L Zhang Q. Chronic exposure to a TLR ligand injures hematopoietic stem cells. J Immunol. (2011) 186:5367–75. doi: 10.4049/jimmunol.1003438 PMC3086167 21441445

[312] Matatall KA Jeong M Chen S Sun D Chen F Mo Q. Chronic infection depletes hematopoietic stem cells through stress-induced terminal differentiation. Cell Rep. (2016) 17:2584–95. doi: 10.1016/j.celrep.2016.11.031 PMC5161248 27926863

[313] MacNamara KC Racine R Chatterjee M Borjesson D Winslow GM. Diminished hematopoietic activity associated with alterations in innate and adaptive immunity in a mouse model of human monocytic ehrlichiosis. Infect Immun. (2009) 77:4061–69. doi: 10.1128/IAI.01550-08 PMC2738008 19451243

[314] Isringhausen S Mun Y Kovtonyuk L Kräutler NJ Suessbier U Gomariz A. Chronic viral infections persistently alter marrow stroma and impair hematopoietic stem cell fitness. J Exp Med. (2021) 218:e20192070. doi: 10.1084/jem.20192070 34709350 PMC8558839

[315] Li F Liu X Niu H Lv W Han X Zhang Y. Persistent stimulation with Mycobacterium tuberculosis antigen impairs the proliferation and transcriptional program of hematopoietic cells in bone marrow. Mol Immunol. (2019) 112:115–22. doi: 10.1016/j.molimm.2019.05.001 31082645

[316] Li F Ma Y Li X Zhang D Han J Tan D. Severe persistent mycobacteria antigen stimulation causes lymphopenia through impairing hematopoiesis. Front Cell Infect Microbiol. (2023) 13:1079774. doi: 10.3389/fcimb.2023.1079774 36743311 PMC9889370

[317] Barreyro L Chlon TM Starczynowski DT. Chronic immune response dysregulation in MDS pathogenesis. Blood. (2018) 132:1553–60. doi: 10.1182/blood-2018-03-784116 PMC6182269 30104218

[318] Feng X Xu H Yin L Yin D Jiang Y. CD4+ T-cell subsets in aplastic anemia, myelodysplastic syndrome, and acute myelogenous leukemia patients: a comparative analysis. Clin Lab. (2023) 69:1477–83. doi: 10.7754/Clin.Lab.2023.221220 37436393

[319] Paracatu LC Monlish DA Greenberg ZJ Fisher DAC Walter MJ Oh ST. Toll-like receptor and cytokine expression throughout the bone marrow differs between patients with low- and high-risk myelodysplastic syndromes. Exp Hematol. (2022) 110:47–59. doi: 10.1016/j.exphem.2022.03.011 35367529 PMC9590644

[320] Greiner J Götz M Hofmann S Schrezenmeier H Wiesneth M Bullinger L. Specific T-cell immune responses against colony-forming cells including leukemic progenitor cells of AML patients were increased by immune checkpoint inhibition. Cancer Immunol Immunother. (2020) 69:629–40. doi: 10.1007/s00262-020-02490-2 PMC11027801 32020256

[321] Camacho V Kuznetsova V Welner RS. Inflammatory cytokines shape an altered immune response during myeloid Malignancies. Front Immunol. (2021) 12:772408. doi: 10.3389/fimmu.2021.772408 34804065 PMC8595317

[322] Zhong FM Yao FY Liu J Zhang HB Li MY Jiang JY. Inflammatory response mediates cross-talk with immune function and reveals clinical features in acute myeloid leukemia. Biosci Rep. (2022) 42:BSR20220647. doi: 10.1042/BSR20220647 35441668 PMC9093697

[323] Guram K Kim SS Wu V Sanders PD Patel S Schoenberger SP. A threshold model for T-cell activation in the era of checkpoint blockade immunotherapy. Front Immunol. (2019) 10:491. doi: 10.3389/fimmu.2019.00491 30936880 PMC6431643

[324] Nakao S Gale RP. Are mild/moderate acquired idiopathic aplastic anaemia and low-risk myelodysplastic syndrome one or two diseases or both and how should it/they be treated? Leukemia. (2016) 30:2127–30. doi: 10.1038/leu.2016.206 27585953

[325] Yamazaki H Nakao S. Border between aplastic anemia and myelodysplastic syndrome. Int J Hematol. (2013) 97:558–63. doi: 10.1007/s12185-013-1324-x 23613266

[326] Goto M Kuribayashi K Takahashi Y Kondoh T Tanaka M Kobayashi D. Identification of autoantibodies expressed in acquired aplastic anaemia. Br J Haematol. (2013) 160:359–62. doi: 10.1111/bjh.12116 23116149

[327] Takamatsu H Feng X Chuhjo T Lu X Sugimori C Okawa K. Specific antibodies to moesin, a membrane-cytoskeleton linker protein, are frequently detected in patients with acquired aplastic anemia. Blood. (2007) 109:2514–20. doi: 10.1182/blood-2006-07-036715 17110458

[328] Hirano N Butler MO Guinan EC Nadler LM Kojima S. Presence of anti- kinectin and anti-PMS1 antibodies in Japanese aplastic anaemia patients. Br J Haematol. (2005) 28:221–3. doi: 10.1111/j.1365-2141.2004.05317.x 15638857

[329] Qi Z Takamatsu H Espinoza JL Lu X Sugimori N Yamazaki H. Autoantibodies specific to hnRNP K: a new diagnostic marker for immune pathophysiology in aplastic anemia. Ann Hematol. (2010) 89:1255–63. doi: 10.1007/s00277-010-1020-3 20623123

[330] Lakota J Lanz A Dubrovcakova M Jankovicova B Gonzalez A Stern M. Antibodies against carbonic anhydrase in patients with aplastic anemia. Acta Haematol. (2012) 128:190–4. doi: 10.1159/000338826 22889718

[331] Hao S Zhang Y Hua L Xie N Xiao N Wang H. Antibodies specific to ferritin light chain polypeptide are frequently detected in patients with immune-related pancytopenia. Mol Med Rep. (2020) 22:2012–20. doi: 10.3892/mmr.2020.11280 PMC7411336 32705249

[332] Takamatsu H Espinoza JL Lu X Qi Z Okawa K Nakao S. Anti-moesin antibodies in the serum of patients with aplastic anemia stimulate peripheral blood mononuclear cells to secrete TNF-alpha and IFN-gamma. J Immunol. (2009) 182:703–10. doi: 10.4049/jimmunol.182.1.703 19109204

[333] Kanchan K Loughran TP Jr. Antigen-driven clonal T cell expansion in disorders of hematopoiesis. Leuk Res. (2003) 27:291–2. doi: 10.1016/s0145-2126(02)00270-9 12531218

[334] Skipper HE Perry S. Kinetics of normal and leukemic leukocyte populations and relevance to chemotherapy. Cancer Res. (1970) 30:1883–97. 4917694

[335] Tsurusawa M Aoyama M Saeki K Fujimoto T. Cell cycle kinetics in childhood acute leukemia studied with in *vitro* bromodeoxyuridine labeling, Ki67-reactivity, and flow cytometry. Leukemia. (1995) 9:1921–5. 7475284

[336] Raza A Alvi S Broady-Robinson L Showel M Cartlidge J Mundle SD. Cell cycle kinetic studies in 68 patients with myelodysplastic syndromes following intravenous iodo- and/or bromodeoxyuridine. Exp Hematol. (1997) 25:530–5. 9197332

[337] Akinduro O Weber TS Ang H Haltalli MLR Ruivo N Duarte D. Proliferation dynamics of acute myeloid leukaemia and haematopoietic progenitors competing for bone marrow space. Nat Commun. (2018) 9:519. doi: 10.1038/s41467-017-02376-5 29410432 PMC5802720

[338] Kumar B Garcia M Weng L Jung X Murakami JL Hu X. Acute myeloid leukemia transforms the bone marrow niche into a leukemia-permissive microenvironment through exosome secretion. Leukemia. (2018) 32:575–87. doi: 10.1038/leu.2017.259 PMC5843902 28816238

[339] Cui P Zhang Y Cui M Li Z Ma G Wang R. Leukemia cells impair normal hematopoiesis and induce functionally loss of hematopoietic stem cells through immune cells and inflammation. Leuk Res. (2018) 65:49–54. doi: 10.1016/j.leukres.2018.01.002 29306107

[340] Zhang TY Dutta R Benard B Zhao F Yin R Majeti R. IL-6 blockade reverses bone marrow failure induced by human acute myeloid leukemia. Sci Transl Med. (2020) 12:eaax5104. doi: 10.1126/scitranslmed.aax5104 32269167 PMC7266679

[341] Su S Liu Z Wang F Zhang Y Chu Y Jiang M. Aplastic anemia associated with Crohn’s disease: a tertiary center retrospective study. Ann Hematol. (2019) 98:2053–61. doi: 10.1007/s00277-019-03729-4 31256218

[342] Shao Y Qi W Zhang X Ran N Liu C Fu R. Plasma metabolomic and intestinal microbial analyses of patients with severe aplastic anemia. Front Cell Dev Biol. (2021) 9:669887. doi: 10.3389/fcell.2021.669887 34497802 PMC8419359

[343] Nakagawa N Maruyama H Zaimoku Y Maruyama K Saito C Katagiri T. Evidence for a common immune pathophysiology in acquired aplastic anemia and ulcerative colitis. Blood. (2015) 126:2418. doi: 10.1182/blood.V126.23.2418.2418

[344] Hisamatsu T Kanai T Mikami Y Yoneno K Matsuoka K Hibi T. Immune aspects of the pathogenesis of inflammatory bowel disease. Pharmacol Ther. (2013) 137:283–97. doi: 10.1016/j.pharmthera.2012.10.008 23103332

[345] Gomez-Bris R Saez A Herrero-Fernandez B Rius C Sanchez-Martinez H Gonzalez-Granado JM. CD4 T-cell subsets and the pathophysiology of inflammatory bowel disease. Int J Mol Sci. (2023) 24:2696. doi: 10.3390/ijms24032696 36769019 PMC9916759

[346] Salmeron G Patey N de Latour RP Raffoux E Gluckman E Brousse N. Coeliac disease and aplastic anaemia: a specific entity? Br J Haematol. (2009) 146:122–4. doi: 10.1111/j.1365-2141.2009.07719.x 19438483

[347] Tokar B Aydoğdu S Paşaoğlu O Ilhan H Kasapoğlu E. Neutropenic enterocolitis: is it possible to break vicious circle between neutropenia and the bowel wall inflammation by surgery? Int J Colorectal Dis. (2003) 18:455–8. doi: 10.1007/s00384-003-0502-3 12750931

[348] Zhao XC Zhao L Sun XY Xu ZS Ju B Meng FJ. Excellent response of severe aplastic anemia to treatment of gut inflammation: A case report and review of the literature. World J Clin cases. (2020) 8:425–35. doi: 10.12998/wjcc.v8.i2.425 PMC7000934 32047795

[349] Zhao XC Xue CJ Song H Gao BH Han FS Xiao SX. Bowel inflammatory presentations on computed tomography in adult patients with severe aplastic anemia during flared inflammatory episodes. World J Clin cases. (2023) 11:576–97. doi: 10.12998/wjcc.v11.i3.0000 PMC9923849 36793625

[350] Kathamuthu GR Sridhar R Baskaran D Babu S. Dominant expansion of CD4+, CD8+ T and NK cells expressing Th1/Tc1/Type 1 cytokines in culture-positive lymph node tuberculosis. PloS One. (2022) 17:e0269109. doi: 10.1371/journal.pone 35617254 PMC9135291

[351] Akter S Chauhan KS Dunlap MD Choreño-Parra JA Lu L Esaulova E. Mycobacterium tuberculosis infection drives a type I IFN signature in lung lymphocytes. Cell Rep. (2022) 39:110983. doi: 10.1016/j.celrep.2022.110983 35732116 PMC9616001

[352] Raviglione MC Snider DE Jr Kochi A. Global epidemiology of tuberculosis. Morbidity and mortality of a worldwide epidemic. JAMA. (1995) 273:220–26. 7807661

[353] Dye C Scheele S Dolin P Pathania V Raviglione MC. Consensus statement. Global burden of tuberculosis: estimated incidence, prevalence, and mortality by country. WHO Global Surveillance and Monitoring Project. JAMA. (1999) 282:677–86. doi: 10.1001/jama.282.7.677 10517722

[354] Houben RM Dodd PJ. The global burden of latent tuberculosis infection: A re-estimation using mathematical modelling. PloS Med. (2016) 13:e1002152. doi: 10.1371/journal.pmed.1002152 27780211 PMC5079585

[355] Carabalí-Isajar ML Rodríguez-Bejarano OH Amado T Patarroyo MA Izquierdo MA Lutz JR. Clinical manifestations and immune response to tuberculosis. World J Microbiol Biotechnol. (2023) 39:206. doi: 10.1007/s11274-023-03636-x 37221438 PMC10205569

[356] Tufariello JM Chan J Flynn JL. Latent tuberculosis: mechanisms of host and bacillus that contribute to persistent infection. Lancet Infect Dis. (2003) 3:578–90. doi: 10.1016/s1473-3099(03)00741-2 12954564

[357] Veatch AV Kaushal D. Opening pandora’s box: mechanisms of mycobacterium tuberculosis resuscitation. Trends Microbiol. (2018) 26:145–57. doi: 10.1016/j.tim.2017.08.001 PMC5794633 28911979

[358] Adekambi T Ibegbu CC Kalokhe AS Yu T Ray SM Rengarajan J. Distinct effector memory CD4+ T cell signatures in latent Mycobacterium tuberculosis infection, BCG vaccination and clinically resolved tuberculosis. PloS One. (2012) 7:e36046. doi: 10.1371/journal.pone.0036046 22545156 PMC3335801

[359] Serbina NV Flynn JL. CD8(+) T cells participate in the memory immune response to Mycobacterium tuberculosis. Infect Immun. (2001) 69:4320–8. doi: 10.1128/IAI.69.7.4320-4328.2001 PMC98502 11401969

[360] Rozot V Vigano S Mazza-Stalder J Idrizi E Day CL Perreau M. Mycobacterium tuberculosis-specific CD8+ T cells are functionally and phenotypically different between latent infection and active disease. Eur J Immunol. (2013) 43:1568–77. doi: 10.1002/eji.201243262 PMC6535091 23456989

[361] Venkatasubramanian S Cheekatla S Paidipally P Tripathi D Welch E Tvinnereim AR. IL-21-dependent expansion of memory-like NK cells enhances protective immune responses against Mycobacterium tuberculosis. Mucosal Immunol. (2017) 10:1031–42. doi: 10.1038/mi.2016.105 PMC5462891 27924822

[362] Garand M Goodier M Owolabi O Donkor S Kampmann B Sutherland JS. Functional and Phenotypic Changes of Natural Killer Cells in Whole Blood during Mycobacterium tuberculosis Infection and Disease. Front Immunol. (2018) 9:257. doi: 10.3389/fimmu.2018.00257 29520269 PMC5827559

[363] Comeau K Paradis P Schiffrin EL. Human and murine memory γδ T cells: Evidence for acquired immune memory in bacterial and viral infections and autoimmunity. Cell Immunol. (2020) 357:104217. doi: 10.1016/j.cellimm.2020.104217 32979762 PMC9533841

[364] Zufferey C Germano S Dutta B Ritz N Curtis N. The contribution of non-conventional T cells and NK cells in the mycobacterial-specific IFNγ response in Bacille Calmette-Guérin (BCG)-immunized infants. PloS One. (2013) 8:e77334. doi: 10.1371/journal.pone.0077334 24098583 PMC3789697

[365] Flores-Gonzalez J Ramón-Luing LA Romero-Tendilla J Urbán-Solano A Cruz-Lagunas A Chavez-Galan L. Latent tuberculosis patients have an increased frequency of IFN-γ-producing CD5+ B cells, which respond efficiently to mycobacterial proteins. Pathogens. (2023) 12:818. doi: 10.3390/pathogens12060818 37375508 PMC10304593

[366] Esmail H Barry CE 3rd Young DB Wilkinson RJ. The ongoing challenge of latent tuberculosis. Philos Trans R Soc Lond B Biol Sci. (2014) 369:20130437. doi: 10.1098/rstb.2013.0437 24821923 PMC4024230

[367] Cadena AM Fortune SM Flynn JL. Heterogeneity in tuberculosis. Nat Rev Immunol. (2017) 17:691–702. doi: 10.1038/nri.2017.69 28736436 PMC6247113

[368] Demiroğlu H Ozcebe OI Ozdemir L Sungur A Dündar S. Pancytopenia with hypocellular bone marrow due to miliary tuberculosis: an unusual presentation. Acta Haematol. (1994) 91:49–51. doi: 10.1159/000204246 8171938

[369] Rutovitz JJ. Miliary tuberculosis causing pancytopenia. A report of 2 cases. S Afr Med J. (1986) 69:451–2. 3961639

[370] Zubair AB Razzaq MT Hashmi AW Ali SMY Israr MM Sadiq SM. Clinical characteristics and etiological spectrum of pancytopenia in pediatric age group: A cross-sectional outlook from a developing country. Cureus. (2022) 14:e27842. doi: 10.7759/cureus.27842 36110464 PMC9462587

[371] Dunphy L Keating E Parke T. Miliary tuberculosis in an immunocompetent male with a fatal outcome. BMJ Case Rep. (2016) 2016:bcr2016216720. doi: 10.1136/bcr-2016-216720 PMC5129105 27807020

[372] Long HJ. Aplastic anemia, a rare complication of disseminated BCG infection: case report. Mil Med. (1982) 147:1067–70. doi: 10.1093/milmed/147.12.1067 6817203

[373] Tahir M Nida A Qamar S. Hiding in the bone: a case of miliary tuberculosis with bone marrow involvement. AME Case Rep. (2018) 2:36. doi: 10.21037/acr.2018.06.05 30264032 PMC6155595

[374] Wipperman MF Bhattarai SK Vorkas CK Maringati VS Taur Y Mathurin L. Gastrointestinal microbiota composition predicts peripheral inflammatory state during treatment of human tuberculosis. Nat Commun. (2021) 12:1141. doi: 10.1038/s41467-021-21475-y 33602926 PMC7892575

[375] Huang SF Yang YY Chou KT Fung CP Wang FD Su WJ. Systemic proinflammation after Mycobacterium tuberculosis infection was correlated to the gut microbiome in HIV-uninfected humans. Eur J Clin Invest. (2019) 49:e13068. doi: 10.1111/eci.13068 30620398

[376] Kim HC Goo JM Kim HB Lee JW Seo JB Im JG. Tuberculosis in patients with myelodysplastic syndromes. Clin Radiol. (2002) 57:408–14. doi: 10.1053/crad.2001.0742 12014940

[377] Al-Anazi KA Al-Jasser AM Evans DA. Infections caused by mycobacterium tuberculosis in patients with hematological disorders and in recipients of hematopoietic stem cell transplant, a twelve year retrospective study. Ann Clin Microbiol Antimicrob. (2007) 6:16. doi: 10.1186/1476-0711-6-16 18021401 PMC2200647

[378] Ganzel C Silverman B Chemtob D Ben Shoham A Wiener-Well Y. The risk of tuberculosis in cancer patients is greatest in lymphoma and myelodysplastic syndrome/myeloproliferative neoplasm: a large population-based cohort study. Leuk Lymphoma. (2019) 60:720–25. doi: 10.1080/10428194.2018.1499904 30188229

[379] Starshinova A Malkova A Kudryavtsev I Kudlay D Zinchenko Y Yablonskiy P. Tuberculosis and autoimmunity: Common features. Tuberculosis (Edinb). (2022) 134:102202. doi: 10.1016/j.tube.2022.102202 35430551

[380] Contini S Pallante M Vejbaesya S Park MH Chierakul N Kim HS. A model of phenotypic susceptibility to tuberculosis: deficient in silico selection of Mycobacterium tuberculosis epitopes by HLA alleles. Sarcoidosis Vasc Diffuse Lung Dis. (2008) 25:21–8. 19070257

[381] Baral T Kurian SJ Thomas L Udyavara Kudru C Mukhopadhyay C Saravu K. Impact of tuberculosis disease on human gut microbiota: a systematic review. Expert Rev Anti Infect Ther. (2023) 21:175–88. doi: 10.1080/14787210.2023.2162879 36564016

[382] Silva F Enaud R Creissen E Henao-Tamayo M Delhaes L Izzo A. Mouse subcutaneous BCG vaccination and mycobacterium tuberculosis infection alter the lung and gut microbiota. Microbiol Spectr. (2022) 10:e0169321. doi: 10.1128/spectrum.01693-21 35652642 PMC9241886

[383] Yamashita M Iwama A. Aging and clonal behavior of hematopoietic stem cells. Int J Mol Sci. (2022) 23:1948. doi: 10.3390/ijms23041948 35216063 PMC8878540

[384] Kurosawa S Iwama A. Aging and leukemic evolution of hematopoietic stem cells under various stress conditions. Inflammation Regener. (2020) 40:29. doi: 10.1186/s41232-020-00138-3 PMC7643313 33292805

[385] Florez MA Tran BT Wathan TK DeGregori J Pietras EM King KY. Clonal hematopoiesis: Mutation-specific adaptation to environmental change. Cell Stem Cell. (2022) 29:882–904. doi: 10.1016/j.stem.2022.05.006 35659875 PMC9202417

[386] Gale RP. Can immune therapy cure acute myeloid leukemia? Curr Treat Options Oncol. (2023) 24:381–86. doi: 10.1007/s11864-023-01066-3 PMC10122615 36949279

[387] Cho DS Schmitt RE Dasgupta A Ducharme AM Doles JD. Acute and sustained alteration to the bone marrow immune microenvironment following polymicrobial infection. Shock. (2022) 58:45–55. doi: 10.1097/SHK.0000000000001951 35984760

[388] Sun XY Yang XD Yang XQ Ju B Xiu NN Xu J. Antibiotic and glucocorticoid-induced recapitulated hematological remission in acute myeloid leukemia: A case report and review of literature. World J Clin cases. (2022) 10:7890–8. doi: 10.12998/wjcc.v10.i22.7890 PMC9372864 36158489

